# Machine learning for medical imaging: methodological failures and recommendations for the future

**DOI:** 10.1038/s41746-022-00592-y

**Published:** 2022-04-12

**Authors:** Gaël Varoquaux, Veronika Cheplygina

**Affiliations:** 1 INRIA, Versailles, France; 2 McGill University, Montreal, Canada; 3 Mila, Montreal, Canada; 4 IT University of Copenhagen, Copenhagen, Denmark

**Keywords:** Computer science, Research data, Medical research

## Abstract

Research in computer analysis of medical images bears many promises to improve patients’ health. However, a number of systematic challenges are slowing down the progress of the field, from limitations of the data, such as biases, to research incentives, such as optimizing for publication. In this paper we review roadblocks to developing and assessing methods. Building our analysis on evidence from the literature and data challenges, we show that at every step, potential biases can creep in. On a positive note, we also discuss on-going efforts to counteract these problems. Finally we provide recommendations on how to further address these problems in the future.

## Introduction

Machine learning, the cornerstone of today’s artificial intelligence (AI) revolution, brings new promises to clinical practice with medical images^1–3^. For example, to diagnose various conditions from medical images, machine learning has been shown to perform on par with medical experts^4^. Software applications are starting to be certified for clinical use^5,6^. Machine learning may be the key to realizing the vision of AI in medicine sketched several decades ago^7^.

The stakes are high, and there is a staggering amount of research on machine learning for medical images. But this growth does not inherently lead to clinical progress. The higher volume of research could be aligned with the academic incentives rather than the needs of clinicians and patients. For example, there can be an oversupply of papers showing state-of-the-art performance on benchmark data, but no practical improvement for the clinical problem. On the topic of machine learning for COVID, Robert et al.^8^ reviewed 62 published studies, but found none with potential for clinical use.

In this paper, we explore avenues to improve clinical impact of machine learning in medical imaging. After sketching the situation, documenting uneven progress in Section It’s not all about larger datasets, we study a number of failures frequent in medical imaging papers, at different steps of the “publishing lifecycle”: what data to use (Section Data, an imperfect window on the clinic), what methods to use and how to evaluate them (Section Evaluations that miss the target), and how to publish the results (Section Publishing, distorted incentives). In each section, we first discuss the problems, supported with evidence from previous research as well as our own analyses of recent papers. We then discuss a number of steps to improve the situation, sometimes borrowed from related communities. We hope that these ideas will help shape research practices that are even more effective at addressing real-world medical challenges.

## It’s not all about larger datasets

The availability of large labeled datasets has enabled solving difficult machine learning problems, such as natural image recognition in computer vision, where datasets can contain millions of images. As a result, there is widespread hope that similar progress will happen in medical applications, algorithm research should eventually solve a clinical problem posed as discrimination task. However, medical datasets are typically smaller, on the order of hundreds or thousands:^9^ share a list of sixteen “large open source medical imaging datasets”, with sizes ranging from 267 to 65,000 subjects. Note that in medical imaging we refer to the number of subjects, but a subject may have multiple images, for example, taken at different points in time. For simplicity here we assume a diagnosis task with one image/scan per subject.

**Fig. 1:**
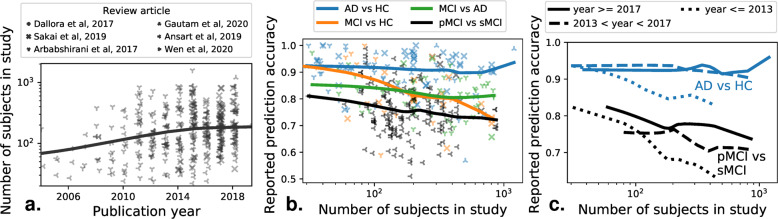
Larger brain-imaging datasets are not enough for better machine-learning diagnosis of Alzheimer’s. A meta-analysis across 6 review papers, covering more than 500 individual publications. The machine-learning problem is typically formulated as distinguishing various related clinical conditions, Alzheimer’s Disease (AD), Healthy Control (HC), and Mild Cognitive Impairment, which can signal prodromal Alzheimer’s . Distinguishing progressive mild cognitive impairment (pMCI) from stable mild cognitive impairment (sMCI) is the most relevant machine-learning task from the clinical standpoint. **a** Reported sample size as a function of the publication year of a study. **b** Reported prediction accuracy as a function of the number of subjects in a study. **c** Same plot distinguishing studies published in different years.

Few clinical questions come as well-posed discrimination tasks that can be naturally framed as machine-learning tasks. But, even for these, larger datasets have to date not lead to the progress hoped for. One example is that of early diagnosis of Alzheimer’s disease (AD), which is a growing health burden due to the aging population. Early diagnosis would open the door to early-stage interventions, most likely to be effective. Substantial efforts have acquired large brain-imaging cohorts of aging individuals at risk of developing AD, on which early biomarkers can be developed using machine learning^10^. As a result, there have been steady increases in the typical sample size of studies applying machine learning to develop computer-aided diagnosis of AD, or its predecessor, mild cognitive impairment. This growth is clearly visible in publications, as on Fig. 1a, a meta-analysis compiling 478 studies from 6 systematic reviews^4^^,^^11–15^.

However, the increase in data size (with the largest datasets containing over a thousand subjects) did not come with better diagnostic accuracy, in particular for the most clinically relevant question, distinguishing pathological versus stable evolution for patients with symptoms of prodromal Alzheimer’s (Fig. 1b). Rather, studies with larger sample sizes tend to report worse prediction accuracy. This is worrisome, as these larger studies are closer to real-life settings. On the other hand, research efforts across time did lead to improvements even on large, heterogeneous cohorts (Fig. 1c), as studies published later show improvements for large sample sizes (statistical analysis in Supplementary Information). Current medical-imaging datasets are much smaller than those that brought breakthroughs in computer vision. Although a one-to-one comparison of sizes cannot be made, as computer vision datasets have many classes with high variation (compared to few classes with less variation in medical imaging), reaching better generalization in medical imaging may require assembling significantly larger datasets, while avoiding biases created by opportunistic data collection, as described below.

## Data, an imperfect window on the clinic

### Datasets may be biased: reflect an application only partly

Available datasets only partially reflect the clinical situation for a particular medical condition, leading to dataset bias^16^. As an example, a dataset collected as part of a population study might have different characteristics that people who are referred to the hospital for treatment (higher incidence of a disease). As the researcher may be unaware of the corresponding dataset bias is can lead to important that shortcomings of the study. Dataset bias occurs when the data used to build the decision model (the training data), has a different distribution than the data on which it should be applied^17^ (the test data). To assess clinically-relevant predictions, the test data must match the actual target population, rather than be a random subset of the same data pool as the train data, the common practice in machine-learning studies. With such a mismatch, algorithms which score high in benchmarks can perform poorly in real world scenarios^18^. In medical imaging, dataset bias has been demonstrated in chest X-rays^19–21^, retinal imaging^22^, brain imaging^23,24^, histopathology^25^, or dermatology^26^. Such biases are revealed by training and testing a model across datasets from different sources, and observing a performance drop across sources.

There are many potential sources of dataset bias in medical imaging, introduced at different phases of the modeling process^27^. First, a cohort may not appropriately represent the range of possible patients and symptoms, a bias sometimes called *spectrum bias*^28^. A detrimental consequence is that model performance can be overestimated for different groups, for example between male and female individuals^21,26^. Yet medical imaging publications do not always report the demographics of the data.

Imaging devices or procedures may lead to specific measurement biases. A bias particularly harmful to clinically relevant automated diagnosis is when the data capture medical interventions. For instance, on chest X-ray datasets, images for the “pneumothorax” condition sometimes show a chest drain, which is a treatment for this condition, and which would not yet be present before diagnosis^29^. Similar spurious correlations can appear in skin lesion images due to markings placed by dermatologists next to the lesions^30^.

Labeling errors can also introduce biases. Expert human annotators may have systematic biases in the way they assign different labels^31^, and it is seldom possible to compensate with multiple annotators. Using automatic methods to extract labels from patient reports can also lead to systematic errors^32^. For example, a report on a follow-up scan that does not mention previously-known findings, can lead to an incorrect “negative” labels.

### Dataset availability distorts research

**Fig. 2:**
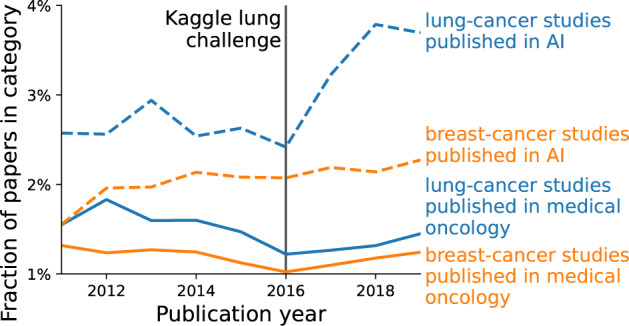
Differences between relative popularity of applications. We show the percentage of papers on lung cancer (in blue) vs breast cancer (in red), relative to all papers within two fields: medical oncology (solid line) and AI (dotted line). Details on how the papers are selected are given in the Supplementary Information). The percentages are relatively constant, except lung cancer in AI, which shows an increase after 2016.

The availability of datasets can influence which applications are studied more extensively. A striking example can be seen in two applications of oncology: detecting lung nodules, and detecting breast tumors in radiological images. Lung datasets are widely available on Kaggle or grand-challenge.org, contrasted with (to our knowledge) only one challenge focusing on mammograms. We look at the popularity of these topics, here defined by the fraction of papers focusing on lung or breast imaging, either in literature on general medical oncology, or literature on AI. In medical oncology this fraction is relatively constant across time for both lung and breast imaging, but in the AI literature lung imaging publications show a substantial increase in 2016 (Fig. 2, methodological details in Supplementary Information). We suspect that the Kaggle lung challenges published around that time contributed to this disproportional increase. A similar point on dataset trends has been made throughout the history of machine learning in general^33^.

### Let us build awareness of data limitations

Addressing such problems arising from the data requires critical thinking about the choice of datasets, at the project level, i.e. which datasets to select for a study or a challenge, and at a broader level, i.e. which datasets we work on as a community.

At the project level, the choice of the dataset will influence the models trained on the data, and the conclusions we can draw from the results. An important step is using datasets from multiple sources, or creating robust datasets from the start when feasible^9^. However, existing datasets can still be critically evaluated for dataset bias^34^, hidden subgroups of patients^29^, or mislabeled instances^35^. A checklist for such evaluation on computer vision datasets is presented in Zendel et al.^18^. When problems are discovered, relabeling a subset of the data can be a worthwhile investment^36^.

At the community level, we should foster understanding of the datasets’ limitations. Good documentation of datasets should describe their characteristics and data collection^37^. Distributed models should detail their limitations and the choices made to train them^38^.

Meta-analyses which look at evolution of dataset use in different areas are another way to reflect on current research efforts. For example, a survey of crowdsourcing in medical imaging^39^ shows a different distribution of applications than surveys focusing on machine learning^1,2^. Contrasting more clinically-oriented venues to more technical venues can reveal opportunities for machine learning research.

## Evaluations that miss the target

### Evaluation error is often larger than algorithmic improvements

Research on methods often focuses on outperforming other algorithms on benchmark datasets. But too strong a focus on benchmark performance can lead to *diminishing returns*, where increasingly large efforts achieve smaller and smaller performance gains. Is this also visible in the development of machine learning in medical imaging?

**Fig. 3:**
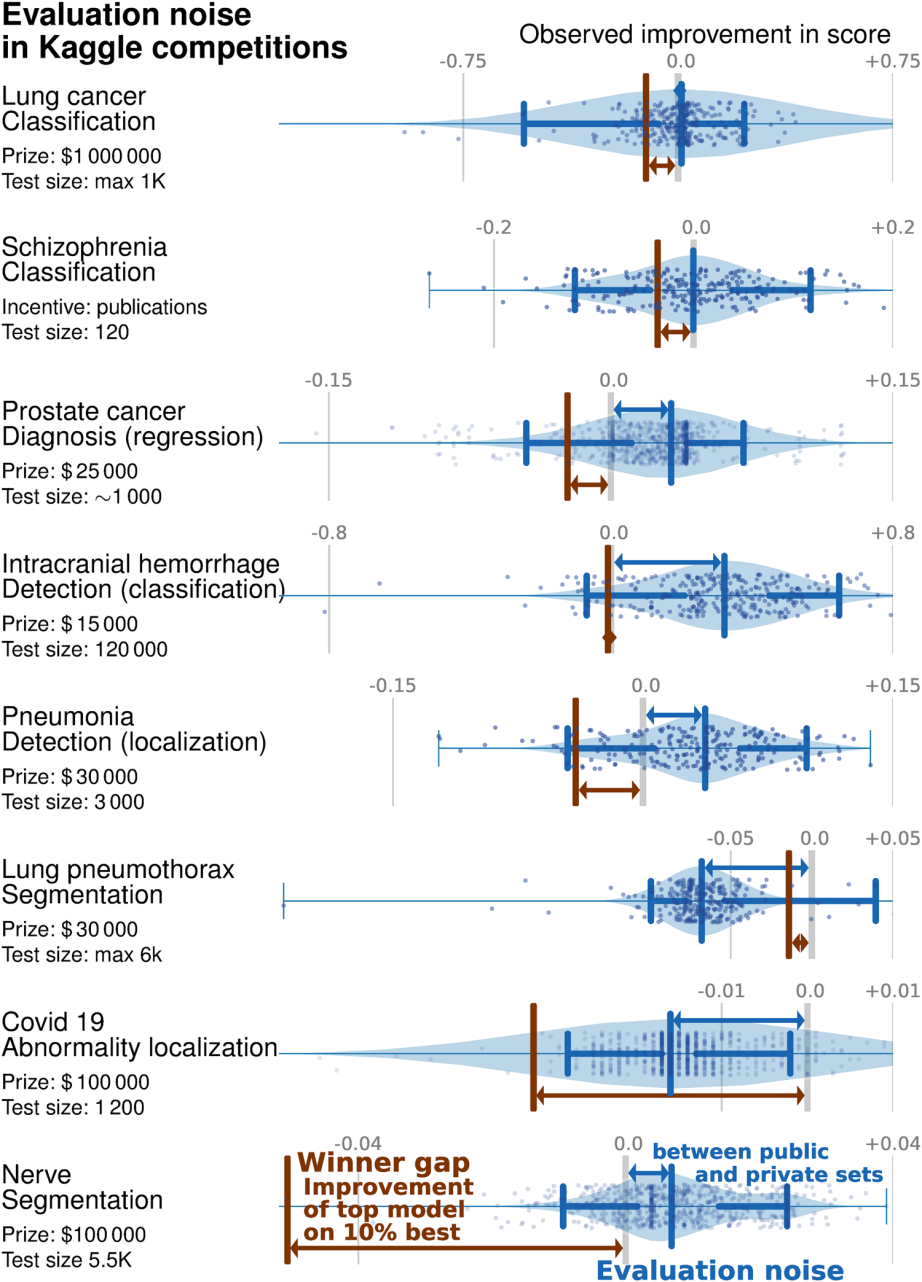
Kaggle challenges: shifts from public to private set compared to improvement across the top 10% models on eight medical-imaging challenges with significant incentives. The blue violin plot shows the *evaluation noise*—the distribution of differences between public and private leaderboards. A systematic shift between public and private set (positive means that the private leaderboard is better than the public leaderboard) indicates overfitting or dataset bias. The width of this distribution shows how noisy the evaluation is, or how representative the public score is for the private score. The brown bar is the *winner gap*, the improvement between the top-most model (the winner) and the 10% best model. It is interesting to compare this improvement to the shift and width in the difference between the public and private sets: if the winner gap is smaller, the 10% best models reached diminishing returns and did not lead to a actual improvement on new data.

We studied performance improvements in 8 Kaggle medical-imaging challenges, 5 on detection of diagnosis of diseases and 3 on image segmentation (details in Supplementary Information). We use the differences in algorithms performance between the public and private leaderboards (two test sets used in the challenge) to quantify the *evaluation noise* –the spread of performance differences between the public and private test sets–, in Fig. 3. We compare its distribution to the *winner gap*—the difference in performance between the best algorithm, and the “top 10%” algorithm.

Overall, 6 of the 8 challenges are in the diminishing returns category. For 5 challenges—lung cancer, schizophrenia, prostate cancer diagnosis and intracranial hemorrhage detection—the evaluation noise is worse than the winner gap. In other words, the gains made by the top 10% of methods are smaller than the expected noise when evaluating a method.

For another challenge, pneumothorax segmentation, the performance on the private set is worse than on the public set, revealing an overfit larger than the winner gap. Only two challenges (covid 19 abnormality and nerve segmentation) display a winner gap larger than the evaluation noise, meaning that the winning method made substantial improvements compared to the 10% competitor.

### Improper evaluation procedures and leakage

Unbiased evaluation of model performance relies on training and testing the models with independent sets of data^40^. However incorrect implementations of this procedure can easily leak information, leading to overoptimistic results. For example some studies classifying ADHD based on brain imaging have engaged in circular analysis^41^, performing feature selection on the full dataset, before cross-validation. Another example of leakage arises when repeated measures of an individual are split across train and test set, the algorithm then learning to recognize the individual patient rather than markers of a condition^42^.

A related issue, yet more difficult to detect, is what we call “overfitting by observer”: even when using cross-validation, overfitting may still occur by the researcher adjusting the method to improve the observed cross-validation performance, which essentially includes the test folds into the validation set of the model. Skocik et al.^43^ provide an illustration of this phenomenon by showing how by adjusting the model this way can lead to better-than-random cross-validation performance for randomly generated data. This can explain some of the overfitting visible in challenges (Section Evaluation error is often larger than algorithmic improvements), though with challenges a private test set reveals the overfitting, which is often not the case for published studies. Another recommendation for challenges would be to hold out several datasets (rather than a part of the same dataset), as is for example done in the Decathlon challenge^44^.

### Metrics that do not reflect what we want

Evaluating models requires choosing a suitable metric. However, our understanding of “suitable” may change over time. For example, an image similarity metric which was widely used to evaluate image registration algorithms, was later shown to be ineffective as scrambled images could lead to high scores^45^.

In medical image segmentation, Maier-Hein et al.^46^ review 150 challenges and show that the typical metrics used to rank algorithms are sensitive to different variants of the same metric, casting doubt on the objectivity of any individual ranking.

Important metrics may be missing from evaluation. Next to typical classification metrics (sensitivity, specificity, area under the curve), several authors argue for a calibration metric that compares the predicted and observed probabilities^28,47^.

Finally, the metrics used may not be synonymous with practical improvement^48,49^. For example, typical metrics in computer vision do not reflect important aspects of image recognition, such as robustness to out-of-distribution examples^49^. Similarly, in medical imaging, improvements in traditional metrics may not necessarily translate to different clinical outcomes, e.g. robustness may be more important than an accurate delineation in a segmentation application.

### Incorrectly chosen baselines

Developing new algorithms builds upon comparing these to baselines. However, if these baselines are poorly chosen, the reported improvement may be misleading.

Baselines may not properly account for recent progress, as revealed in machine-learning applications to healthcare^50^, but also other applications of machine learning^51–53^.

Conversely, one should not forget simple approaches effective for the problem at hand. For example, Wen et al.^14^ show that convolutional neural networks do not outperform support vector machines for Alzheimer’s disease diagnosis from brain imaging.

Finally, minute implementation details of algorithms may be important and many are not aware of implementation factors^54^.

### Statistical significance not tested, or misunderstood

Experimental results are by nature noisy: results may depend on which specific samples were used to train the models, the random initializations, small differences in hyper-parameters^55^. However, benchmarking predictive models currently lacks well-adopted statistical good practices to separate out noise from generalizable findings.

A first, well-documented, source of brittleness arises from machine-learning experiments with too small sample sizes^56^. Indeed, testing predictive modeling requires many samples, more than conventional inferential studies, else the measured prediction accuracy may be a distant estimation of real-life performance. Sample sizes are growing, albeit slowly^57^. On a positive note, a meta-analysis of public vs private leaderboards on Kaggle^58^ suggests that overfitting is less of an issue with “large enough” test data (at least several thousands).

Another challenge is that strong validation of a method requires it to be robust to details of the data. Hence validation should go beyond a single dataset, and rather strive for statistical consensus across multiple datasets^59^. Yet, the corresponding statistical procedures require dozens of datasets to establish significance and are seldom used in practice. Rather, medical imaging research often reuses the same datasets across studies, which raises the risk of finding an algorithm that performs well by chance, in an implicit multiple comparison problem^60^.

But overall medical imaging research seldom analyzes how likely empirical results are to be due to chance: only 6% of segmentation challenges surveyed^61^, and 15% out of 410 popular computer science papers published by ACM used a statistical test^62^.

However, null-hypothesis tests are often misinterpreted^63^, with two notable challenges: (1) the lack of statistically significant results does not demonstrate the absence of effect, and (2) any trivial effect can be significant given enough data^64,65^. For these reasons, Bouthiellier et al.^66^ recommend to replace traditional null-hypothesis testing with *superiority testing*, testing that the improvement is above a given threshold.

### Let us redefine evaluation

#### Higher standards for benchmarking

Good machine-learning benchmarks are difficult. We compile below several recognized best practices for medical machine learning evaluation^28,40,67,68^:

- Safeguarding from data leakage by separating out all test data from the start, before any data transformation.
- A documented way of selecting model hyper-parameters (including architectural parameters for neural networks, the use of additional (unlabeled) dataset or transfer learning^2^), without ever using data from the test set.
- Enough data in the test set to bring statistical power, at least several hundreds samples, ideally thousands or more^9^, and confidence intervals on the reported performance metric—see Supplementary Information. In general, more research on appropriate sample sizes for machine learning studies would be helpful.
- Rich data to represent the diversity of patients and disease heterogeneity, ideally multi-institutional data including all relevant patient demographics and disease state, with explicit inclusion criteria; other cohorts with different recruitment go the extra mile to establish external validity^69,70^.
- Strong baselines that reflect the state of the art of machine-learning research, but also historical solutions including clinical methodologies not necessarily relying on medical imaging.
- A discussion the variability of the results due to arbitrary choices (random seeds) and data sources with an eye on statistical significance—see Supplementary Information.
- Using different quantitative metrics to capture the different aspects of the clinical problem and relating them to relevant clinical performance metrics. In particular, the potential health benefits from a detection of the outcome of interest should be used to choose the right trade off between false detections and misses^71^.
- Adding qualitative accounts and involving groups that will be most affected by the application in the metric design^72^.

#### More than beating the benchmark

Even with proper validation and statistical significance testing, measuring a tiny improvement on a benchmark is seldom useful. Rather, one view is that, beyond rejecting a null, a method should be accepted based on evidence that it brings a sizable improvement upon the existing solutions. This type of criteria is related to *superiority tests* sometimes used in clinical trials^73–75^. These tests are easy to implement in predictive modeling benchmarks, as they amount to comparing the observed improvement to variation of the results due to arbitrary choices such as data sampling or random seeds^55^.

Organizing blinded challenges, with a hidden test set, mitigate the winner’s curse. But to bring progress, challenges should not only focus on the winner. Instead, more can be learned by comparing the competing methods and analyzing the determinants of success, as well as failure cases.

#### Evidence-based medicine good practices

A machine-learning algorithm deployed in clinical practice is a health intervention. There is a well-established practice to evaluate the impact of health intervention, building mostly on randomized clinical trials^76^. These require actually modifying patients’ treatments and thus should be run only after thorough evaluation on historical data.

A solid trial evaluates a well-chosen measure of patient health outcome, as opposed to predictive performance of an algorithm. Many indirect mechanisms may affect this outcome, including how the full care processes adapts to the computer-aided decision. For instance, a positive consequence of even imperfect predictions may be reallocating human resources to complex cases. But a negative consequence may be over-confidence leading to an increase in diagnostic errors. Cluster randomized trials can account for how modifications at the level of care unit impact the individual patient: care units, rather than individuals are randomly allocated to receive the intervention (the machine learning algorithm)^77^. Often, double blind is impossible: the care provider is aware of which arm of the study is used, the baseline condition or the system evaluated. Providers’ expectations can contribute to the success of a treatment, for instance via indirect placebo or nocebo effects^78^, making objective evaluation of the health benefits challenging, if these are small.

## Publishing, distorted incentives

### No incentive for clarity

The publication process does not create incentives for clarity. Efforts to impress may give rise to unnecessary “mathiness” of papers or suggestive language^79^ (such as “human-level performance”).

Important details may be omitted, from ablation experiments showing what part of the method drives improvements^79^, to reporting how algorithms were evaluated in a challenge [46]. This in turn undermines reproducibility: being able to reproduce the exact results or even draw the same conclusions^80,81^.

### Optimizing for publication

As researchers our goal should be to solve scientific problems. Yet, the reality of the culture we exist in can distort this objective. Goodhart’s law summarizes well the problem: *when a measure becomes a target, it ceases to be a good measure*. As our academic incentive system is based publications, it erodes their scientific content via Goodhart’s law.

Methods publication are selected for their novelty. Yet, comparing 179 classifiers on 121 datasets shows no statistically significant differences between the top methods [82]. In order to sustain novelty, researchers may be introducing unnecessary complexity into the methods, that do not improve their prediction but rather contribute to technical debt, making systems harder to maintain and deploy^83^.

Another metric emphasized is obtaining “state-of-the-art” results, which leads to several of the evaluation problems outlined in Section Evaluations that miss the target. The pressure to publish “good” results can aggravate methodological loopholes^84^, for instance gaming the evaluation in machine learning^85^. It is then all too appealing to find after-the-fact theoretical justifications of positive yet fragile empirical findings. This phenomenon, known as *HARKing* (hypothesizing after the results are known)^86^, has been documented in machine learning^87^ and computer science in general^62^.

Finally, the selection of publications creates the so-called “file drawer problem”^88^: positive results, some due to experimental flukes, are more likely to be published than corresponding negative findings. For example, in 410 most downloaded papers from the ACM, 97% of the papers which used significance testing had a finding with *p*-value of less than 0.05^62^. It seems highly unlikely that only 3% of the initial working hypotheses—even for impactful work—turned out not confirmed.

### Let us improve our publication norms

Fortunately there are various alleys to improve reporting and transparency. For instance, the growing set of open datasets could be leveraged for collaborative work beyond the capacities of a single team^89^. The set of metrics studied could then be broadened, shifting the publication focus away from a single-dimension benchmark. More metrics can indeed help understanding a method’s strengths and weaknesses^41,90,91^, exploring for instance calibration metrics^28,47,92^ or learning curves^93^. The medical-research literature has several reporting guidelines for prediction studies^67,94,95^. They underline many points raised in previous sections: reporting on how representative the study sample is, on the separation between train and test data, on the motivation for the choice of outcome, evaluation metrics, and so forth. Unfortunately, algorithmic research in medical imaging seldom refers to these guidelines.

Methods should be studied on more than prediction performance: reproducibility^81^, carbon footprint^96^, or a broad evaluation of costs should be put in perspective with the real-world patient outcomes, from a putative clinical use of the algorithms^97^.

Preregistration or registered reports can bring more robustness and trust: the motivation and experimental setup of a paper are to be reviewed before empirical results are available, and thus the paper is be accepted before the experiments are run^98^. Translating this idea to machine learning faces the challenge that new data is seldom acquired in a machine learning study, yet it would bring sizeable benefits^62,99^.

More generally, accelerating the progress in science calls for accepting that some published findings are sometimes wrong^100^. Popularizing different types of publications may help, for example publishing negative results^101^, replication studies^102^, commentaries^103^ and reflections on the field^68^ or the recent NeurIPS Retrospectives workshops. Such initiatives should ideally be led by more established academics, and be welcoming of newcomers^104^.

## Conclusions

Despite great promises, the extensive research in medical applications of machine learning seldom achieves a clinical impact. Studying the academic literature and data-science challenges reveals troubling trends: accuracy on diagnostic tasks progresses slower on research cohorts that are closer to real-life settings; methods research is often guided by dataset availability rather than clinical relevance; many developments of model bring improvements smaller than the evaluation errors. We have surveyed challenges of clinical machine-learning research that can explain these difficulties. The challenges start with the choice of datasets, plague model evaluation, and are amplified by publication incentives. Understanding these mechanisms enables us to suggest specific strategies to improve the various steps of the research cycle, promoting publications best practices^105^. None of these strategies are silver-bullet solutions. They rather require changing procedures, norms, and goals. But implementing them will help fulfilling the promises of machine-learning in healthcare: better health outcomes for patients with less burden on the care system.

## Supplementary information

Supplementary information

LaTeX source files

## Data Availability

For reproducibility, all data used in our analyses are available on https://github.com/GaelVaroquaux/ml_med_imaging_failures.

## Code availability

For reproducibility, all code for our analyses is available on https://github.com/GaelVaroquaux/ml_med_imaging_failures.
