# Supplementary material for: Machine learning for medical imaging: methodological failures and recommendations for the future
Source: NPJ Digit Med. 2022 Apr 12;5:48. doi: 10.1038/s41746-022-00592-y (PMC9005663; doi:10.1038/s41746-022-00592-y)
Supplement: Supplementary file 1 — Supplementary information [file 41746_2022_592_MOESM1_ESM.pdf]

Supplementary information to: Machine learning for medical imaging: methodological failures and recommendations for the future

Gaël Varoquaux [1,2,3]

Veronika Cheplygina [4]

[1] INRIA, France

[2] McGill University, Montreal, Canada

[3] Mila, Montreal, Canada

[4] IT University of Copenhagen, Denmark

gael.varoquaux@inria.fr, vech@itu.dk

March 1, 2022

Testing procedures for predictive models

There is no one-size-fit-all testing procedures for machine learning classifiers. However, in this section we provide some recommendations on

what to (not) do via an illustrative example.

Suppose we are interested in detecting cancer from lung images. Given a dataset of healthy and cancerous images, and a performance metric of interest –such as accuracy– how can we design statistically-sound evaluation of a classifier? There are several different underlying questions that call for different methods.

Evaluating a prediction rule

The first question that we might be interested in is: given a prediction rule how well does it perform? The prediction rule can be independent of the images, or it can come from the output of a classifier trained on the data. In both settings, evidence for clinical application of the prediction rule, for instance as required by regulatory agencies, calls for statistical evaluation. For evaluating the prediction rule we can use confidence intervals or null-hypothesis testing. For this we need test data, which (in machine learning) is often a held-out part of the existing dataset, or new data –external validation. The size of the test set then determines the statistical power: the confidence errors on the measure of the prediction performance and the effect size that can be detected. The test set should be large enough, for example too small test sets lead to large error bars of the estimated prediction performance.[1] Riley *et al*[2] give recommendations on minimum sample sizes for various performance metrics.

Evaluation of a machine-learning procedure

Another question we might be interested in is to evaluate a machine-learning procedure. Unlike a prediction rule, by *machine-learning procedure* we refer the full process of starting from training data, extracting a prediction rule, and using it to classify test images as healthy or cancerous. This question is often of interest in machine-learning research, or if we want to retrain an existing prediction rule on new data. Here we need different evaluation techniques because the machine-learning procedure has several uncontrolled sources of variance, such as the training set or random initialization.[3] For machine-learning research, conclusions on a given procedure should not be driven by the choice of particularly favorable training set if we cannot expect similar performance when using new training data. On the contrary, for clinical applications, it is safer to evaluate an already trained algorithm which will be used as the prediction rule in practice, to rule out the possibility of a poor performance if training the algorithm on new training data.

Given our dataset of lung images, a good evaluation of a learning procedure requires repeatedly sampling different training and testing data –as in a cross-validation loop–, as well as other sources of variance. Due to the flexibility of machine-learning classifiers, it is hard derive closed-form expressions of confidence intervals or p-values to account for all the sources of variability. Instead, we can estimate the distribution of performance scores by repeating the experiments with such variations and deduce

confidence intervals.[3] Note that standard statistical tests (such as the t-test) cannot be used across cross-validation folds, as these are not independent samples.[1]

Sample size is an important factor to the success of prediction studies, both for the training data and the testing data. To evaluate of much the amount of data impacts the prediction performance, we can use *learning curves* [1] where we vary the training set size, evaluate the trained classifier on the test set, and plot the performance metric as a function of the training set size. If the curve is flattening, we might conclude that adding more training data will not improve performance. We might also be able to observe that less flexible classifiers (such as linear models) might outperform more flexible classifiers (such as neural networks) when the training set is small, but the situation to reverse when more training data is added. To give a concrete example, we refer to the results from a machine-learning paper by one of the authors[4] where classifiers are evaluated on non-medical datasets, but with similar dataset sizes and evaluation metrics as often used in medical imaging. We refer to Fig. 7 in,[4] which is not reproduced here for copyright reasons. This figure shows several panels, each panel corresponding to one benchmark dataset. Each panel shows a learning curve with the training set size on the x-axis, and the area under the curve (AUC, higher is better) on the y-axis, for seven different classifiers. We see

[1]Note that we use the original definition of learning curves where a classifier is trained and evaluated multiple times, rather than the recent trend of referring to the loss of a single training run

AUC increases with the training set size, but the slopes of the classifiers are different. For example, looking at the “Musk1”dataset, we see that the classifier “minimax libsvc” starts out being the worst classifier, but is among the best at larger training sizes. Ideally, this plot should have also included error bars on the performances.

Comparing machine-learning procedures

In machine-learning research we might want to evaluate that a classifier is better than one or more competing classifiers. The question is then whether the difference in the observed performance metrics is due to chance.
Given a particular dataset and a classifier –a learning procedure–, cross-validation can give an estimate of the expected performance and its distribution. But we cannot yet conclude our classifier is better than another classifier for detecting lung cancer in images in general: in particular, we would need to evaluate the classifier on other, independent, datasets.
In this scenario, we can compare *ranks* of classifiers on multiple independent datasets to conclude that a classifier is generally better than another, as recommended by[5] (though with caveats pointed out by the same author[6]). Based on the number of datasets (samples) and the number of classifiers, we can test whether the average classifier ranks are due to chance. If not, we can use a post-hoc test to find the critical difference: the minimum difference in ranks that classifiers need have, to be considered

significantly-different. The critical difference decreases with the number of datasets, but increases with the number of classifiers.

We show an illustration of the evaluation procedure in Table 1, also based on data from results from Cheplygina *et al.*[4] The table shows results for 14 different datasets (rows) and six classifiers (columns). For each dataset/classifier combination, the mean and standard error of the performance metric, which is the area under the curve (AUC), is reported (missing results are due classifiers failing to converge, and are ranked as last). The last row the table shows the average ranks of the classifiers, based on the Friedman test recommended by.[5] Since the null hypothesis (that the differences in these ranks overall are due to chance) is rejected, the critical difference is calculated, which for 14 datasets and six classifiers is equal to 2.0153. From these results we could conclude that although MInD is the classifier with the lowest rank, MILES and Minimax are not significantly different, because their ranks are within the critical difference from 1.7857.

Brain imaging biomarkers meta-analysis

While the sample size of studies increases with time, there is a wide variability. We run a multivariable regression analysis, to separate out the effect of sample size of the study and publication date on reported prediction accuracy. Table 2 gives the corresponding estimated normalized coefficients, confidence intervals, and p-values. It confirms what is visible in

Table 1: Area under the curve (AUC) and standard error (×100), 5 × 10-fold cross-validation for 14 datasets and 6 classifiers. The last row shows the classifier ranks from the Friedman test, for which the critical difference is 2.0153. Classifiers in bold are best, or not significantly worse than best. Reproduced from Cheplygina *et al.*[4]

	Classifier					
Data	emdd	misvm	boosting	miles	minimax	meanmin
Musk1	87.4 (2.1)	81.3 (2.5)	74.3 (2.6)	92.8 (1.2)	89.1 (1.9)	93.4 (1.2)
Musk2	86.9 (2.1)	81.5 (2.1)	73.6 (2.3)	95.3 (0.8)	89.0 (1.5)	95.4 (1.4)
Fox	67.6 (3.2)	53.9 (1.6)	61.1 (1.9)	69.8 (1.7)	58.1 (1.3)	60.5 (1.9)
Tiger	75.4 (2.9)	83.3 (1.3)	84.1 (1.6)	87.2 (1.6)	81.4 (1.3)	85.1 (1.7)
Elephant	88.5 (2.1)	84.1 (1.4)	89.0 (1.4)	88.3 (1.3)	88.2 (1.0)	93.1 (0.8)
African	91.5 (1.0)	63.4 (1.2)	88.9 (0.9)	58.9 (1.7)	84.5 (1.5)	96.7 (0.4)
Beach	84.7 (1.3)	49.6 (1.6)	85.0 (1.1)	60.0 (1.9)	82.4 (0.9)	92.3 (0.6)
AjaxOrange	-	93.6 (1.1)	97.9 (0.5)	-	91.1 (0.9)	98.6 (0.4)
alt.atheism	51.0 (5.2)	70.9 (2.6)	-	47.1 (2.4)	80.6 (1.8)	94.9 (1.0)
comp.graphics	48.2 (3.2)	59.3 (2.8)	56.3 (2.6)	57.2 (2.6)	57.1 (2.7)	92.2 (1.4)
BrownCreeper	94.5 (0.9)	85.8 (0.7)	95.4 (0.4)	95.8 (0.3)	94.1 (0.4)	95.5 (0.3)
WinterWren	98.5 (0.3)	95.3 (0.4)	97.0 (1.5)	99.2 (0.2)	98.1 (0.2)	99.5 (0.1)
Web1	-	89.7	77.8 (5.7)	88.2 (4.7)	90.4	76.0 (2.7)
Web4	60.6 (1.1)	81.2	61.8 (4.9)	70.8 (1.6)	86.7	73.7 (3.2)
Rank	4.1786	4.3571	3.9286	**3.1786**	**3.5714**	**1.7857**

Fig. 1c: for a given publication date, studies with larger sample sizes report lower prediction accuracy. Publication time, on the other hand, is associated with an improvement in prediction accuracy.

That reported prediction accuracy decreased with study sample size has already been reported.[1] There are multiple reasons that can explain such a finding. First, larger cohorts tend to be more heterogeneous, and thus lead to harder prediction tasks. Second, the smaller the cohort, the smaller the test set; as a result, it is more likely that a good prediction accuracy is observed by chance, due to sampling error on the test set. This good prediction accuracy would however be misleading, as it would not reflect an

Table 2: **Regression analysis of published accuracy as a function of sample size and publication year**

	coef	Confidence interval			p value
pMCI vs sMCI ($n = 166$)					
log(subjects)	-0.0352	-0.059	—	-0.012	0.004
Year	0.0074	0.002	—	0.012	0.004
AD vs HC ($n = 86$)					
log(subjects)	-0.0188	-0.037	—	-0.000	0.047
Year	0.0024	-0.002	—	0.006	0.224

actual generalization capacity to new data.

Literature popularity review methods

We give here the methodological details behind Fig. 2. To assess relative popularity of studies on breast versus lung cancer in medical and AI research, we quantify the prevalence of these topics in the corresponding literature. For this, we use the Dimensions.AI app,[7] querying the titles and abstracts of papers, with the following two queries:

- lung AND (tumor OR nodule) AND (scan OR image)
- breast AND (tumor OR nodule) AND (scan OR image)

We do this for two categories, which are the largest subcategories within top-level categories "medical sciences" and "information computing":

- 1112 Oncology and Carcinogenesis
- 0801 Artificial Intelligence and Image Processing

We then normalize the number of papers per year, by the total number of papers for the "cancer AND (scan OR image)" query in the respective categories (1112 Oncology or 0801 AI).

Included Kaggle challenges

We selected 8 medical-imaging challenges from Kaggle, which allows efficient retrieval of public and private leaderboard scores. In July 2021, there were around 15 medical-imaging challenges available, of which we selected four based on their varying focus (classification or segmentation) and incentives. Table 3 gives details on the challenges we use to compare performance gains to evaluation noise.

For each competition, we looked at the public and private leaderboards, extracting the following information:

- Differences d_i, defined by the difference of the i-th algorithm between the public and private leaderboard
- Distribution of d_i's per competition, its mean and standard deviation
- The interval t_{10}, defined by the difference between the best algorithm, and the "top 10%" algorithm

Description	URL	Incentive	Test size	Entries
Lung cancer detection in CT scans	https://www.kaggle.com/c/data-science-bowl-2017	1M USD	max 1K	394
Schizophrenia classification in MR scans	https://www.kaggle.com/c/mlsp-2014-mri/overview	Publications	120	313
Lung pneumothorax segmentation in X-rays	https://www.kaggle.com/c/siim-acr-pneumothorax-segmentation	30K USD	max 6K	350
Nerve segmentation in ultrasound images	https://www.kaggle.com/c/ultrasound-nerve-segmentation	100K USD	5.5K	922
Intracranial hemorrhage detection in CT images	https://www.kaggle.com/c/rsna-intracranial-hemorrhage-detection	15K USD	120K	2 553
Prostate cancer grade assessment	https://www.kaggle.com/c/prostate-cancer-grade-assessment	25K USD	1K	19 723
COVID-19 abnormalities location on chest radiographs	https://www.kaggle.com/c/siim-covid19-detection	100K USD	1 200	32 307
Pneumonia detection from chest radiographs	https://www.kaggle.com/c/rsna-pneumonia-detection-challenge	30K USD	3 00	2 001

Table 3: Details of Kaggle challenges used for our analysis. The test size shows the number of test images provided, and the number of entries corresponds to the number of results on the private leaderboard.

Supplementary References

[1] Varoquaux, G. Cross-validation failure: small sample sizes lead to large error bars. *NeuroImage* **180**, 68–77 (2018).

[2] Riley, R. D. *et al.* Minimum sample size for external validation of a clinical prediction model with a binary outcome. *Statistics in Medicine* (2021).

[3] Bouthillier, X. *et al.* Accounting for variance in machine learning benchmarks. In *Machine Learning and Systems* (2021).

[4] Cheplygina, V., Tax, D. M. J. & Loog, M. Multiple instance learning with bag dissimilarities. *Pattern recognition* **48**, 264–275 (2015).

[5] Demšar, J. Statistical comparisons of classifiers over multiple data sets. *The Journal of Machine Learning Research* **7**, 1–30 (2006).

[6] Demšar, J. On the appropriateness of statistical tests in machine learning. In *ICML workshop on Evaluation Methods for Machine Learning*, 65 (2008).

[7] Mori, A. & Taylor, M. Dimensions metrics API reference & getting started. *Digital Science & Research solutions* (2018).
