# Supplementary material for: Machine learning for medical imaging: methodological failures and recommendations for the future
Source: NPJ Digit Med. 2022 Apr 12;5:48. doi: 10.1038/s41746-022-00592-y (PMC9005663; doi:10.1038/s41746-022-00592-y)
Supplement: Supplementary file 2 — LaTeX source files [file 41746_2022_592_MOESM2_ESM.zip › diff-with-original-submission.pdf]

Machine learning for medical imaging: methodological failures and recommendations for the future

Gaël Varoquaux[*†‡], Veronika Cheplygina[§]
[*]INRIA, France
[†]McGill University, Montreal, Canada
[‡]Mila, Montreal, Canada
[§]IT University of Copenhagen, Denmark

Abstract

Research in computer analysis of medical images bears many promises to improve patients' health. However, a number of systematic challenges are slowing down the progress of the field, from limitations of the data, such as biases, to research incentives, such as optimizing for publication. In this paper we review roadblocks to developing and assessing methods. Building our analysis on evidence from the literature and data challenges, we show that at every step, potential biases can creep in. On a positive note, we also discuss on-going efforts to counteract these problems. Finally we provide recommendations on how to further address these problems in the future.

I. Introduction

Machine learning, the cornerstone of today's artificial intelligence (AI) revolution, brings new promises to clinical practice with medical images [Litjens et al., 2017, Cheplygina et al., 2019, Zhou et al., 2020]. For example, to diagnose various conditions from medical images, machine learning has been shown to perform on par with medical experts [see Liu et al., 2019, for a recent overview]. Software applications are starting to be certified for clinical use [Topol, 2019, Sendak et al., 2020]. Machine learning may be the key to realizing the vision of AI in medicine sketched several decades ago [Schwartz et al., 1987].

The stakes are high, and there is a staggering amount of research on machine learning for medical images. But this growth does not inherently lead to clinical progress. The higher volume of research could be aligned with the academic incentives rather than the needs of clinicians and patients. For example, there can be an oversupply of papers showing state-of-the-art performance on benchmark data, but no practical improvement for the clinical problem. On the topic of machine learning for COVID, Roberts et al. [2021] reviewed 62 published studies, but found none with potential for clinical use.

In this paper, we explore avenues to improve clinical impact of machine learning in medical imaging. After sketching the situation, documenting uneven progress in Section II, we study a number of failures frequent in medical imaging papers, at different steps of the "publishing lifecycle": what data to use (Section III), what methods to use and how to evaluate them (Section IV), and how to publish the results (Section V). In each section we first discuss the problems, supported with evidence from previous research

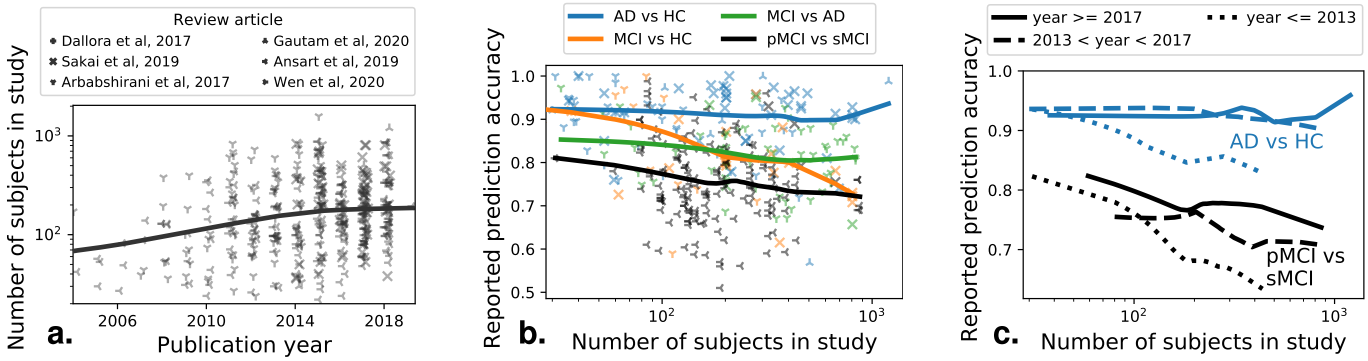

Fig. 1. **Larger brain-imaging datasets are not enough for better machine-learning diagnosis of Alzheimer's**. A meta-analysis across 6 review papers, covering more than 500 individual publications. The machine-learning problem is typically formulated as distinguishing various related clinical conditions, Alzheimer's Disease (AD), Healthy Control (HC), and Mild Cognitive Impairment, which can signal prodromal Alzheimer's. Distinguishing progressive mild cognitive impairment (pMCI) from stable mild cognitive impairment (sMCI) is the most relevant machine-learning task from the clinical standpoint. **a.** Reported sample size as a function of the publication year of a study. **b.** Reported prediction accuracy as a function of the number of subjects in a study. **c.** Same plot distinguishing studies published in different years.

as well as our own analyses of recent papers. We then discuss a number of steps to improve the situation, sometimes borrowed from related communities. We hope that these ideas will help shape research practices that are even more effective at addressing real-world medical challenges.

II. It's not all about larger datasets

The availability of large labeled datasets has enabled solving difficult machine learning problems, such as natural image recognition in computer vision, where datasets can contain millions of images. As a result, there is widespread hope that similar progress will happen in medical applications~~: with large datasets~~, algorithm research should eventually solve a clinical problem posed as discrimination task. However, medical datasets are typically smaller, on the order of hundreds or thousands: Willemink et al. [2020] share a list of sixteen "large open source medical imaging datasets", with sizes ranging from 267 to 65 000 subjects. Note that in medical imaging we refer to the number of subjects, but a subject may have multiple images, for example, taken at different points in time. For simplicity here we assume a diagnosis task with one image/scan per subject.

Few clinical questions come as well-posed discrimination tasks that can be naturally framed as machine-learning tasks. But, even for these, larger datasets have to date not lead to the progress hoped for.

One example is that of early diagnosis of Alzheimer's disease (AD), which is a growing health burden due to the aging population. Early diagnosis would open the door to early-stage interventions, most likely to be effective. Substantial efforts have acquired large brain-imaging cohorts of aging individuals at risk of developing AD, on which early biomarkers can be developed using machine learning [Mueller et al., 2005]. As a result, there have been steady increases in the typical sample size of studies applying machine learning to develop computer-aided diagnosis of AD, or its predecessor, mild cognitive impairment. This growth is clearly visible in publications, as on Figure 1a, a meta-analysis compiling 478 studies from 6 systematic reviews [Dallora et al., 2017, Arbabshirani et al., 2017, Liu et al., 2019, Sakai and Yamada, 2019, Wen et al., 2020, Ansart et al., 2020].

However, the increase in data size (with the largest datasets containing over a thousand subjects) did not come with better diagnostic accuracy, in particular for the most clinically relevant question, distinguishing pathological versus stable evolution for patients with symptoms of prodromal Alzheimer's (Figure 1b). Rather, studies with larger sample sizes tend to report worse prediction accuracy. This is worrisome, as these larger studies are closer to real-life settings. On the other hand, research efforts across time did lead to improvements even on large, heterogeneous cohorts (Figure 1c), as studies published later show improvements for large sample sizes (statistical analysis in appendix B). Current medical-imaging datasets are much smaller than those that brought breakthroughs in computer vision. Reaching new regimes Although a one-to-one comparison of sizes cannot be made, as computer vision datasets have many classes with high variation (compared to few classes with less variation in medical imaging), reaching better generalization in medical imaging may require assembling significantly larger datasets, while avoiding biases created by opportunistic data collection, as described below.

III. Data, an imperfect window on the clinic

A. *Datasets may be biased: reflect an application only partly*

Available datasets only partially reflect the clinical situation for a particular medical condition, leading to dataset bias Torralba and Efros [2011][Torralba and Efros, 2011]. As an example, a dataset collected as part of a population study might have different characteristics that people who are referred to the hospital for treatment (higher incidence of a disease). The As the researcher may be unaware of the corresponding dataset bias is all the more important that the researcher may be unaware itcan lead to important that shortcomings of the study. Dataset bias occurs when the data used to build the decision model (the training data), has a different distribution than the data on which it should be applied (the test data) [Dockès et al., 2021]. To assess clinically-relevant predictions, the test data must match the actual target population, rather than be a random subset of the same data pool as the train data, the common practice in machine-learning studies. With such a mismatch, algorithms which score high in benchmarks can perform poorly in real world scenarios [Zendel et al., 2017]. In medical imaging, dataset bias has been demonstrated in chest X-rays [Pooch et al., 2019, Zech et al., 2018, Larrazabal et al., 2020], retinal imaging [Tasdizen et al., 2018], brain imaging [Wachinger et al., 2021, Ashraf et al., 2018], histopathology [Yu et al., 2018], or dermatology [Abbasi-Sureshjani et al., 2020]. Such biases are revealed by training and testing a model across datasets from different sources, and observing a performance drop across sources.

There are many potential sources of dataset bias in medical imaging, introduced at different phases of the modeling process [Suresh and Guttag, 2019]. First, a cohort may not appropriately represent the range of possible patients and symptoms, a bias sometimes called *spectrum bias* [Park and Han, 2018]. A detrimental consequence is that model performance can be overestimated for different groups, for example between male and female individuals [Abbasi-Sureshjani et al., 2020, Larrazabal et al., 2020]. Yet medical imaging publications do not always report the demographics of the data.

Imaging devices or procedures may lead to specific measurement biases. A bias particularly harmful to clinically relevant automated diagnosis is when the data capture medical interventions. For instance, on chest X-ray datasets, images for the "pneumothorax" condition sometimes show a chest drain, which is a treatment for this condition, and which would not yet be present before diagnosis [Oakden-Rayner

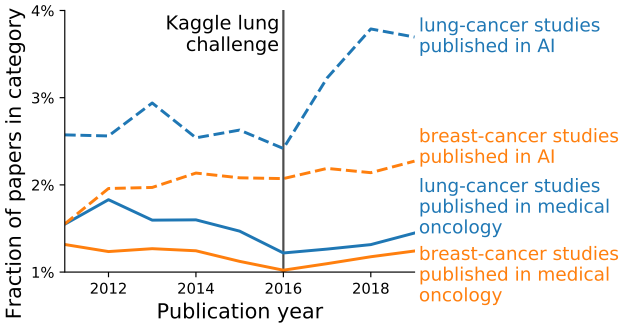

Fig. 2. **Differences between relative popularity of applications.** We show the percentage of papers on lung cancer (in blue) vs breast cancer (in red), relative to all papers within two fields: medical oncology (solid line) and AI (dotted line). Details on how the papers are selected are given in Supp. Mat. C). The percentages are relatively constant, except lung cancer in AI, which shows an increase after 2016.

et al., 2020]. Similar spurious correlations can appear in skin lesion images due to markings placed by dermatologists next to the lesions [Winkler et al., 2019].

Labeling errors can also introduce biases. Expert human annotators may have systematic biases in the way they assign different labels [Joskowicz et al., 2019], and it is seldom possible to compensate with multiple annotators. Using automatic methods to extract labels from patient reports can also lead to systematic errors [Oakden-Rayner, 2020]. For example, a report on a follow-up scan that does not mention previously-known findings, can lead to an incorrect "negative" labels.

B. *Dataset availability distorts research*

The availability of datasets can influence which applications are studied more extensively. A striking example can be seen in two applications of oncology: detecting lung nodules, and detecting breast tumors in radiological images. Lung datasets are widely available on Kaggle or grand-challenge.org, contrasted with (to our knowledge) only one challenge focusing on mammograms. We look at the popularity of these topics, here defined by the fraction of papers focusing on lung or breast imaging, either in literature on general medical oncology, or literature on AI. In medical oncology this fraction is relatively constant across time for both lung and breast imaging, but in the AI literature lung imaging publications show a substantial increase in 2016 (Figure 2, methodological details in Supp. Mat. C). We suspect that the Kaggle lung challenges published around that time contributed to this disproportional increase. A similar point on dataset trends has been made throughout the history of machine learning in general [Langley, 2011].

C. *Let us build awareness of data limitations*

Addressing such problems arising from the data requires critical thinking about the choice of datasets, at the project level, i.e. which datasets to select for a study or a challenge, and at a broader level, i.e. which datasets we work on as a community.

At the project level, the choice of the dataset will influence the models trained on the data, and the conclusions we can draw from the results. An important step is using datasets from multiple sources, or creating robust datasets from the start when feasible [Willemink et al., 2020]. However, existing datasets can still be critically evaluated for dataset bias [Rabanser et al., 2018], hidden subgroups of patients [Oakden-Rayner et al., 2020], or mislabeled instances [Rädsch et al., 2020]. A checklist for such evaluation on computer vision datasets is presented in Zendel et al. [2017]. When problems are discovered, relabeling a subset of the data can be a worthwhile investment [Beyer et al., 2020].

At the community level, we should foster understanding of the datasets' limitations. Good documentation of datasets should describe their characteristics and data collection [Gebru et al., 2018]. Distributed models should detail their limitations and the choices made to train them [Mitchell et al., 2019].

Meta-analyses which look at evolution of dataset use in different areas are another way to reflect on current research efforts. For example, a survey of crowdsourcing in medical imaging [Ørting et al., 2020] shows a different distribution of applications than surveys focusing on machine learning [Litjens et al., 2017, Cheplygina et al., 2019]. Contrasting more clinically-oriented venues to more technical venues can reveal opportunities for machine learning research.

IV. Evaluations that miss the target

A. *Evaluation error is often larger than algorithmic improvements*

Research on methods often focuses on outperforming other algorithms on benchmark datasets. But too strong a focus on benchmark performance can lead to *diminishing returns*, where increasingly large efforts achieve smaller and smaller performance gains. Is this also visible in the development of machine learning in medical imaging?

We studied performance improvements in ~~four~~ 8 Kaggle medical-imaging challenges, ~~two on disease classification and two~~ 5 on detection of diagnosis of diseases and 3 on image segmentation (details in Supp. Mat. D). We use the differences in algorithms performance between the public and private leaderboards (two test sets used in the challenge) to quantify the *evaluation noise* –the spread of performance differences between the public and private test sets–, in Figure 3. We compare its distribution to *winner gap* the difference in performance between the best algorithm, and the "top 10%" algorithm.

Overall, ~~three of the four~~ 6 of the 8 challenges are in the diminishing returns category. For ~~two challenges , schizophrenia and lung cancer diagnosis~~ 5 challenges –lung cancer, schizophrenia, prostate cancer diagnosis and intracranial hemorrhage detection–, the evaluation noise is worse than the winner gap. In other words, the gains made by the top 10% of methods are smaller than the expected noise when evaluating a method.

For ~~a third~~ another challenge, pneumothorax segmentation, the performance on the private set is worse than on the public set, revealing an overfit larger than the winner gap. Only ~~the nerve segmentationchallenge displays~~ two challenge (covid 19 abnormality and nerve segmentation) display a winner gap ~~smaller~~ larger than the evaluation noise, meaning that the winning method made substantial improvements compared to the 10% competitor.

B. *Improper evaluation procedures and leakage*

Unbiased evaluation of model performance relies on training and testing the models with independent sets of data [Poldrack et al., 2020]. However incorrect implementations of this procedure can easily leak information, leading to overoptimistic results. For example some studies classifying ADHD based on brain imaging have engaged in circular analysis [Pulini et al., 2019], performing feature selection on the full dataset, before cross-validation. Another example of leakage arises when repeated measures of an individual are split across train and test set, the algorithm then learning to recognize the individual patient rather than markers of a condition [Saeb et al., 2017].

Lung cancer
Classification
Prize: $1 000 000Test size: max 1K
Schizophrenia
Classification
Incentive: publicationsTest size: 120
Lung pneumothorax
Segmentation
Prize: $30 000Test size: max 6k
Nerve
Segmentation
Prize: $100 000Test size

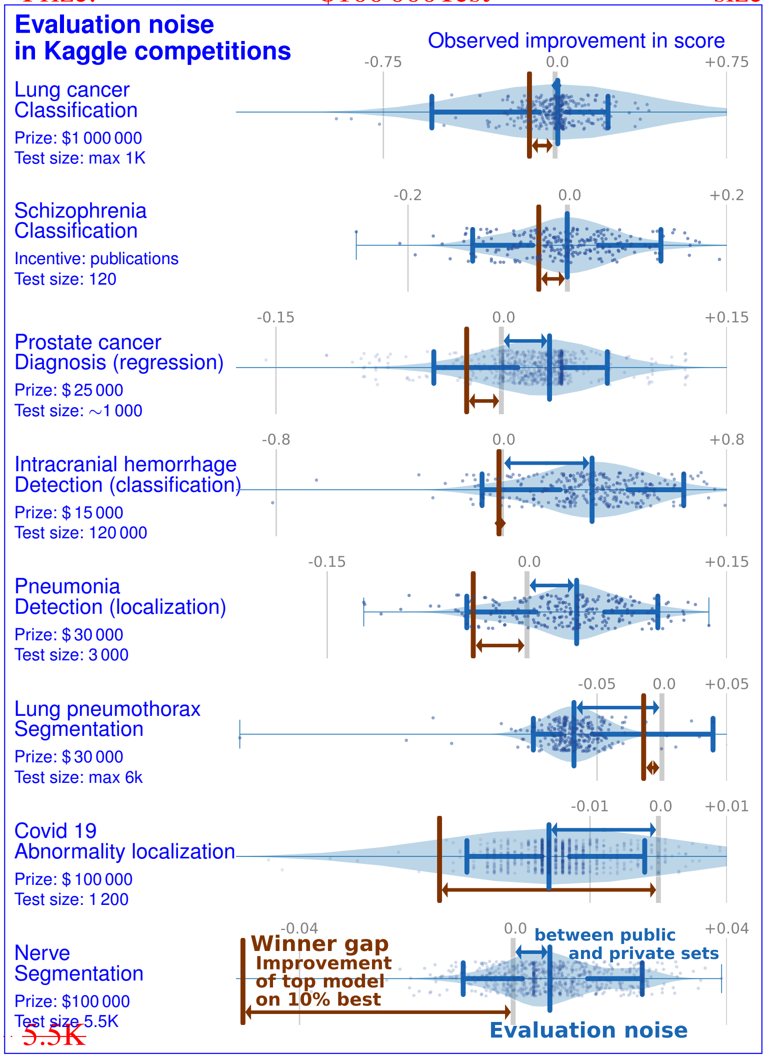

Fig. 3. **Kaggle challenges: shifts from public to private set compared to improvement across the top 10% models** on 4 medical-imaging challenges with significant incentives. The blue violin plot shows the *evaluation noise* – the distribution of differences between public and private leaderboards. A systematic shift between public and private set (positive means that the private leaderboard is better than the public leaderboard) indicates overfitting or dataset bias. The width of this distribution shows how noisy the evaluation is, or how representative the public score is for the private score. The brown bar is the *winner gap*, the improvement between the top-most model (the winner) and the 10% best model. It is interesting to compare this improvement to the shift and width in the difference between the public and private sets: if the winner gap is smaller, the 10% best models reached diminishing returns and did not lead to a actual improvement on new data.

A related issue, yet more difficult to detect, is what we call “overfitting by observer”. Even when : even when using cross-validationis carried out for all steps of the method, overfitting may still occur by the researcher adjusting the method to improve the observed cross-validation performance[Hosseini et al., 2020], which essentially includes the test folds into the validation set of the model. Hosseini et al. [2020] provide a great illustration of this phenomenon by showing how by adjusting the model this way can lead to better-than-random cross-validation performance for randomly generated data. This can explain some of

the overfitting visible in challenges (Section IV-A), though with challenges a private test set reveals the overfitting, which is often not the case for published studies. Another recommendation for challenges would be to hold out several datasets (rather than a part of the same dataset), as is for example done in the Decathlon challenge ~~Simpson et al. [2019]~~[Simpson et al., 2019].

C. Metrics that do not reflect what we want

Evaluating models requires choosing a suitable metric. However, our understanding of "suitable" may change over time. For example, an image similarity metric which was widely used to evaluate image registration algorithms, was later shown to be ineffective as scrambled images could lead to high scores [Rohlfing, 2011].

In medical image segmentation, Maier-Hein et al. [2018a] review 150 challenges and show that the typical metrics used to rank algorithms are sensitive to different variants of the same metric, casting doubt on the objectivity of any individual ranking.

Important metrics may be missing from evaluation. Next to typical classification metrics (sensitivity, specificity, area under the curve), several authors argue for a calibration metric that compares the predicted and observed probabilities [Park and Han, 2018, Van Calster et al., 2019].

Finally, the metrics used may not be synonymous with practical improvement [Wagstaff, 2012, Shankar et al., 2020]. For example, typical metrics in computer vision do not reflect important aspects of image recognition, such as robustness to out-of-distribution examples [Shankar et al., 2020]. Similarly, in medical imaging, improvements in traditional metrics may not necessarily translate to different clinical outcomes, e.g. robustness may be more important than an accurate delineation in a segmentation application.

D. Incorrectly chosen baselines

Developing new algorithms builds upon comparing these to baselines. However, if these baselines are poorly chosen, the reported improvement may be misleading.

Baselines may not properly account for recent progress, as revealed in machine-learning applications to healthcare [Bellamy et al., 2020], but also other applications of machine learning [Oliver et al., 2018, Dacrema et al., 2019, Musgrave et al., 2020].

Conversly, one should not forget simple approaches effective for the problem at hand. For example, Wen et al. [2020] show that convolutional neural networks do not outperform support vector machines for Alzheimer's disease diagnosis from brain imaging.

Finally, minute implementation details of algorithms may be important and many are not aware of implementation factors [Pham et al., 2020].

E. Statistical significance not tested, or misunderstood

Experimental results are by nature noisy: results may depend on which specific samples were used to train the models, the random initializations, small differences in hyper-parameters [Bouthillier et al., 2021]. However, benchmarking predictive models currently lacks well-adopted statistical good practices to separate out noise from generalizable findings.

A first, well-documented, source of brittleness arises from machine-learning experiments with too small sample sizes [Varoquaux, 2018]. Indeed, testing predictive modeling requires many samples, more than conventional inferential studies, else the measured prediction accuracy may be a distant estimation of real-life performance. Sample sizes are growing, albeit slowly [Szucs and Ioannidis, 2020]. On a positive note, a meta-analysis of public vs private leaderboards on Kaggle [Roelofs et al., 2019] suggests that overfitting is less of an issue with “large enough” test data (at least several thousands).

Another challenge is that strong validation of a method requires it to be robust to details of the data. Hence validation should go beyond a single dataset, and rather strive for statistical consensus across multiple datasets [Demšar, 2006]. Yet, the corresponding statistical procedures require dozens of datasets to establish significance and are seldom used in practice. Rather, medical imaging research often reuses the same datasets across studies, which raises the risk of finding an algorithm that performs well by chance, in an implicit multiple comparison problem [Thompson et al., 2020].

But overall medical imaging research seldom analyzes how likely empirical results are to be due to chance: only 6% of segmentation challenges surveyed [Maier-Hein et al., 2018b], and 15% out of 410 popular computer science papers published by ACM used a statistical test [Cockburn et al., 2020].

However, null-hypothesis tests are often misinterpreted [Gigerenzer, 2018], with two notable challenges: *1)* the lack of statistically significant results does not demonstrate the absence of effect, and *2)* any trivial effect can be significant given enough data [Benavoli et al., 2016, Berrar, 2017]. For these reasons, Bouthillier et al. [2019] recommend to replace traditional null-hypothesis testing by *superiority testing*, testing that the improvement is above a given threshold.

F. Let us redefine evaluation

a) Higher standards for benchmarking: Good machine-learning benchmarks are difficult. We compile below several recognized best practices for medical machine learning evaluation [Park and Han, 2018, Poldrack et al., 2020, Norgeot et al., 2020, Drummond, 2006]:

- Safeguarding from data leakage by separating out all test data from the start, before any data transformation.
- A documented way of selecting model hyper-parameters (including architectural parameters for neural networks, the use of additional (unlabeled) dataset or transfer learning [Cheplygina et al., 2019]), without ever using data from the test set.
- Enough data in the test set to bring statistical power, at least several hundreds samples, ideally thousands or more [Willemink et al., 2020], and confidence intervals on the reported performance metric – see Appendix A1. In general, more research on appropriate sample sizes for machine learning studies would be helpful.
- Rich data to represent the diversity of patients and disease heterogeneity, ideally multi-institutional data including all relevant patient demographics and disease state, with explicit inclusion criteria; other cohorts with different recruitment go the extra mile to establish external validity [Steyerberg and Harrell, 2016, Woo et al., 2017].
- Strong baselines that reflect the state of the art of machine-learning research, but also historical solutions including clinical methodologies not necessarily relying on medical imaging.

- A discussion the variability of the results due to arbitrary choices (random seeds) and data sources with an eye on statistical significance – see Appendix A2.
- Using different quantitative metrics to capture the different aspects of the clinical problem and relating them to relevant clinical performance metrics. In particular, the potential health benefits from a detection of the outcome of interest should be used to choose the right trade off between false detections and misses [Van Calster et al., 2018].
- Adding qualitative accounts and involving groups that will be most affected by the application in the metric design [Thomas and Uminsky, 2020].

b) More than beating the benchmark: Even with proper validation and statistical significance testing, measuring a tiny improvement on a benchmark is seldom useful. Rather, one view is that, beyond rejecting a null, a method should be accepted based on evidence that it brings a sizable improvement upon the existing solutions. This type of criteria is related to *superiority tests* sometimes used in clinical trials [for the Evaluation of Medicinal Products, 2001, D'Agostino Sr et al., 2003, Christensen, 2007]. These tests are easy to implement in predictive modeling benchmarks, as they amount to comparing the observed improvement to variation of the results due to arbitrary choices such as data sampling or random seeds [Bouthillier et al., 2021].

Organizing blinded challenges, with a hidden test set, mitigate the winner's curse. But to bring progress, challenges should not only focus on the winner. Instead, more can be learned by comparing the competing methods and analyzing the determinants of success, as well as failure cases.

c) Evidence-based medicine good practices: A machine-learning algorithm deployed in clinical practice is a health intervention. There is a well-established practice to evaluate the impact of health intervention, building mostly on randomized clinical trials [see eg "impact and Implementation" in Hendriksen et al., 2013]. These require actually modifying patients' treatments and thus should be run only after thorough evaluation on historical data.

A solid trial evaluates a well-chosen measure of patient health outcome, as opposed to predictive performance of an algorithm. Many indirect mechanisms may affect this outcome, including how the full care processes adapts to the computer-aided decision. For instance, a positive consequence of even imperfect predictions may be reallocating human resources to complex cases. But a negative consequence may be over-confidence leading to an increase in diagnostic errors. Cluster randomized trials can account for how modifications at the level of care unit impact the individual patient: care units, rather than individuals are randomly allocated to receive the intervention (the machine learning algorithm) [Campbell et al., 2004]. Often, double blind is impossible: the care provider is aware of which arm of the study is used, the baseline condition or the system evaluated. Providers' expectations can contribute to the success of a treatment, for instance via indirect placebo or nocebo effects [Blasini et al., 2018], making objective evaluation of the health benefits challenging, if these are small.

V. Publishing, distorted incentives

A. No incentive for clarity

The publication process does not create incentives for clarity. Efforts to impress may give rise to unnecessary "mathiness" of papers or suggestive language (such as "human-level performance") [Lipton

and Steinhardt, 2019].

Important details may be omitted, from ablation experiments showing what part of the method drives improvements [Lipton and Steinhardt, 2019], to reporting how algorithms were evaluated in a challenge [Maier-Hein et al., 2018a]. This in turn undermines reproducibility: being able to reproduce the exact results or even draw the same conclusions [Tatman et al., 2018, Gundersen and Kjensmo, 2018].

B. *Optimizing for publication*

As researchers our goal should be to solve scientific problems. Yet, the reality of the culture we exist in can distort this objective. Goodhart's law summarizes well the problem: *when a measure becomes a target, it ceases to be a good measure*. As our academic incentive system is based publications, it erodes their scientific content via Goodhart's law.

Methods publication are selected for their novelty. Yet, comparing 179 classifiers on 121 datasets shows no statistically significant differences between the top methods [Fernández-Delgado et al., 2014]. In order to sustain novelty, researchers may be introducing unnecessary complexity into the methods, that do not improve their prediction but rather contribute to technical debt, making systems harder to maintain and deploy [Sculley et al., 2015].

Another metric emphasized is obtaining "state-of-the-art" results, which leads to several of the evaluation problems outlined in Section IV. The pressure to publish "good" results can aggravate methodological loopholes [Ioannidis, 2005], for instance gaming the evaluation in machine learning [Teney et al., 2020]. It is then all too appealing to find after-the-fact theoretical justifications of positive yet fragile empirical findings. This phenomenon, known as *HARKing* (hypothesizing after the results are known) [Kerr, 1998], has been documented in machine learning [Gencoglu et al., 2019] and computer science in general [Cockburn et al., 2020].

Finally, the selection of publications creates the so-called "file drawer problem" [Rosenthal, 1979]: positive results, some due to experimental flukes, are more likely to be published than corresponding negative findings. For example, in 410 most downloaded papers from the ACM, 97% of the papers which used significance testing had a finding with p-value of less than 0.05 [Cockburn et al., 2020]. It seems highly unlikely that only 3% of the initial working hypotheses –even for impactful work– turned out not confirmed.

C. *Let us improve our publication norms*

Fortunately there are various alleys to improve reporting and transparency. For instance, the growing set of open datasets could be leveraged for collaborative work beyond the capacities of a single team [Kellmeyer, 2017]. The set of metrics studied could then be broadened, shifting the publication focus away from a single-dimension benchmark. More metrics can indeed help understanding a method's strengths and weaknesses [Japkowicz and Shah, 2015, Santafe et al., 2015, Pulini et al., 2019], exploring for instance calibration metrics [Park and Han, 2018, Han et al., 2016, Van Calster et al., 2019] or learning curves [Richter and Khoshgoftaar, 2020]. The medical-research literature has several reporting guidelines for prediction studies [Collins et al., 2015, Wolff et al., 2019, Norgeot et al., 2020]. They underline many points raised in previous sections: reporting on how representative the study sample is, on the

separation between train and test data, on the motivation for the choice of outcome, evaluation metrics... Unfortunately, algorithmic research in medical imaging seldom refers to these guidelines.

Methods should be studied on more than prediction performance: reproducibility [Gundersen and Kjensmo, 2018], carbon footprint [Henderson et al., 2020], or a broad evaluation of costs should be put in perspective with the real-world patient outcomes, from a putative clinical use of the algorithms [Bowen and Casadevall, 2015].

Preregistration or registered reports can bring more robustness and trust: the motivation and experimental setup of a paper are to be reviewed before empirical results are available, and thus the paper is be accepted before the experiments are run [Chambers et al., 2015]. Translating this idea to machine learning faces the challenge that new data is seldom acquired in a machine learning study, yet it would bring sizeable benefits [Forde and Paganini, 2019, Cockburn et al., 2020].

More generally, accelerating the progress in science calls for accepting that some published findings are sometimes wrong [Firestein, 2015]. Popularizing different types of publications may help, for example publishing negative results [Borji, 2018], replication studies [Voets et al., 2018], commentaries [Wilkinson et al., 2020] and reflections on the field such as [Drummond, 2006] or the recent NeurIPS Retrospectives workshops. Such initiatives should ideally be led by more established academics, and be welcoming of newcomers [Whitaker and Guest, 2020].

VI. Conclusions

Despite great promises, the extensive research in medical applications of machine learning seldom achieves a clinical impact. Studying the academic literature and data-science challenges reveals troubling trends: accuracy on diagnostic tasks progresses slower on research cohorts that are closer to real-life settings; methods research is often guided by dataset availability rather than clinical relevance; many developments of model bring improvements smaller than the evaluation errors. We have surveyed challenges of clinical machine-learning research that can explain these difficulties. The challenges start with the choice of datasets, plague model evaluation, and are amplified by publication incentives. Understanding these mechanisms enables us to suggest specific strategies to improve the various steps of the research cycle, promoting publications best practices [Kakarmath et al., 2020]. None of these strategies are silver-bullet solutions. They rather require changing procedures, norms, and goals. But implementing them will help fulfilling the promises of machine-learning in healthcare: better health outcomes for patients with less burden on the care system.

Data availability

For reproducibility, all data used in our analyses are available on https://github.com/GaelVaroquaux/ml_med_imaging_failures.

Code availability

For reproducibility, all code for our analyses is available on https://github.com/GaelVaroquaux/ml_med_imaging_failures.

Competing Interests

The authors declare that there are no competing interests.

Author Contribution

Both VC and GV collected the data; conceived, designed, and performed the analysis; reviewed the literature; and wrote the paper.

Acknowledgements

We would like to thank Alexandra Elbakyan for help with the literature review. We thank Pierre Dragicevic for providing feedback on early versions of this manuscript, and Pierre Bartet for comments on the preprint. We also thank the reviewers, Jack Wilkinson and ~~the anonymous reviewer~~Odd Erik Gundersen, for excellent comments which improved our manuscript.

GV acknowledges funding from grant ANR-17-CE23-0018, DirtyData.

References

S. Abbasi-Sureshjani, R. Raumanns, B. E. Michels, G. Schouten, and V. Cheplygina. Risk of training diagnostic algorithms on data with demographic bias. In *Interpretable and Annotation-Efficient Learning for Medical Image Computing*, pages 183–192. Springer, 2020.

M. Ansart, S. Epelbaum, G. Bassignana, A. Bône, S. Bottani, T. Cattai, R. Couronne, J. Faouzi, I. Koval, M. Louis, et al. Predicting the progression of mild cognitive impairment using machine learning: a systematic, quantitative and critical review. *Medical Image Analysis*, page 101848, 2020.

M. R. Arbabshirani, S. Plis, J. Sui, and V. D. Calhoun. Single subject prediction of brain disorders in neuroimaging: Promises and pitfalls. *NeuroImage*, 145:137–165, 2017.

A. Ashraf, S. Khan, N. Bhagwat, M. Chakravarty, and B. Taati. Learning to unlearn: building immunity to dataset bias in medical imaging studies. In *NeurIPS workshop on Machine Learning for Health (ML4H)*. 2018.

D. Bellamy, L. Celi, and A. L. Beam. Evaluating progress on machine learning for longitudinal electronic healthcare data. *arXiv preprint arXiv:2010.01149*, 2020.

A. Benavoli, G. Corani, and F. Mangili. Should we really use post-hoc tests based on mean-ranks? *The Journal of Machine Learning Research*, 17(1):152–161, 2016.

D. Berrar. Confidence curves: an alternative to null hypothesis significance testing for the comparison of classifiers. *Machine Learning*, 106(6):911–949, 2017.

L. Beyer, O. J. Hénaff, A. Kolesnikov, X. Zhai, and A. v. d. Oord. Are we done with ImageNet? *arXiv preprint arXiv:2006.07159*, 2020.

M. Blasini, N. Peiris, T. Wright, and L. Colloca. The role of patient–practitioner relationships in placebo and nocebo phenomena. *International review of neurobiology*, 139:211–231, 2018.

A. Borji. Negative results in computer vision: A perspective. *Image and Vision Computing*, 69:1–8, 2018.

X. Bouthillier, C. Laurent, and P. Vincent. Unreproducible research is reproducible. In *International Conference on Machine Learning (ICML)*, pages 725–734, 2019.

X. Bouthillier, P. Delaunay, M. Bronzi, A. Trofimov, B. Nichyporuk, J. Szeto, N. Mohammadi Sepahvand, E. Raff, K. Madan, V. Voleti, S. E. Kahou, V. Michalski, T. Arbel, C. Pal, G. Varoquaux, and P. Vincent. Accounting for variance in machine learning benchmarks. In *Machine Learning and Systems*, 2021.

A. Bowen and A. Casadevall. Increasing disparities between resource inputs and outcomes, as measured by certain health deliverables, in biomedical research. *Proceedings of the National Academy of Sciences*, 112(36):11335–11340, 2015.

M. K. Campbell, D. R. Elbourne, and D. G. Altman. Consort statement: extension to cluster randomised trials. *Bmj*, 328(7441):702–708, 2004.

C. D. Chambers, Z. Dienes, R. D. McIntosh, P. Rotshtein, and K. Willmes. Registered reports: realigning incentives in scientific publishing. *Cortex*, 66:A1–A2, 2015.

V. Cheplygina, D. M. J. Tax, and M. Loog. Multiple instance learning with bag dissimilarities. *Pattern recognition*, 48(1):264–275, 2015.

V. Cheplygina, M. de Bruijne, and J. P. W. Pluim. Not-so-supervised: a survey of semi-supervised, multi-instance, and transfer learning in medical image analysis. *Medical Image Analysis*, 54:280–296, 2019.

E. Christensen. Methodology of superiority vs. equivalence trials and non-inferiority trials. *Journal of Hepatology*, 46(5):947–954, 2007.

A. Cockburn, P. Dragicevic, L. Besançon, and C. Gutwin. Threats of a replication crisis in empirical computer science. *Communications of the ACM*, 63(8):70–79, 2020.

G. S. Collins, J. B. Reitsma, D. G. Altman, and K. G. Moons. Transparent reporting of a multivariable prediction model for individual prognosis or diagnosis (tripod): the tripod statement. *Journal of British Surgery*, 102(3):148–158, 2015.

M. F. Dacrema, P. Cremonesi, and D. Jannach. Are we really making much progress? a worrying analysis of recent neural recommendation approaches. In *ACM Conference on Recommender Systems*, pages 101–109, 2019.

R. B. D'Agostino Sr, J. M. Massaro, and L. M. Sullivan. Non-inferiority trials: design concepts and issues–the encounters of academic consultants in statistics. *Statistics in Medicine*, 22(2):169–186, 2003.

A. L. Dallora, S. Eivazzadeh, E. Mendes, J. Berglund, and P. Anderberg. Machine learning and microsimulation techniques on the prognosis of dementia: A systematic literature review. *PLoS ONE*, 12(6):e0179804, 2017.

J. Demšar. Statistical comparisons of classifiers over multiple data sets. *The Journal of Machine Learning Research*, 7:1–30, 2006.

J. Demšar. On the appropriateness of statistical tests in machine learning. In *ICML workshop on Evaluation Methods for Machine Learning*, page 65, 2008.

J. Dockès, G. Varoquaux, and J.-B. Poline. Preventing dataset shift from breaking machine-learning biomarkers. *GigaScience*, 10(9):giab055, 2021.

C. Drummond. Machine learning as an experimental science (revisited). In *AAAI workshop on evaluation methods for machine learning*, pages 1–5, 2006.

M. Fernández-Delgado, E. Cernadas, S. Barro, D. Amorim, and D. Amorim Fernández-Delgado. Do we need hundreds of classifiers to solve real world classification problems? *Journal of Machine Learning*

Research, 15:3133–3181, 2014. ISSN 1532-4435.

S. Firestein. *Failure: Why science is so successful.* Oxford University Press, 2015.

E. A. for the Evaluation of Medicinal Products. Points to consider on switching between superiority and non-inferiority. *British Journal of Clinical Pharmacology*, 52(3):223–228, 2001.

J. Z. Forde and M. Paganini. The scientific method in the science of machine learning. In *ICLR workshop on Debugging Machine Learning Models*. 2019.

T. Gebru, J. Morgenstern, B. Vecchione, J. W. Vaughan, H. M. Wallach, H. D. III, and K. Crawford. Datasheets for datasets. In *Workshop on Fairness, Accountability, and Transparency in Machine Learning*. 2018.

O. Gencoglu, M. van Gils, E. Guldogan, C. Morikawa, M. Süzen, M. Gruber, J. Leinonen, and H. Huttunen. HARK side of deep learning–from grad student descent to automated machine learning. *arXiv preprint arXiv:1904.07633*, 2019.

G. Gigerenzer. Statistical rituals: The replication delusion and how we got there. *Advances in Methods and Practices in Psychological Science*, 1(2):198–218, 2018.

O. E. Gundersen and S. Kjensmo. State of the art: Reproducibility in artificial intelligence. In *AAAI Conference on Artificial Intelligence*, 2018.

K. Han, K. Song, and B. W. Choi. How to develop, validate, and compare clinical prediction models involving radiological parameters: study design and statistical methods. *Korean Journal of Radiology*, 17(3):339–350, 2016.

P. Henderson, J. Hu, J. Romoff, E. Brunskill, D. Jurafsky, and J. Pineau. Towards the systematic reporting of the energy and carbon footprints of machine learning. *Journal of Machine Learning Research*, 21 (248):1–43, 2020.

J. M. Hendriksen, G.-J. Geersing, K. G. Moons, and J. A. de Groot. Diagnostic and prognostic prediction models. *Journal of Thrombosis and Haemostasis*, 11:129–141, 2013.

M. Hosseini, M. Powell, J. Collins, C. Callahan-Flintoft, W. Jones, H. Bowman, and B. Wyble. I tried a bunch of things: The dangers of unexpected overfitting in classification of brain data. *Neuroscience & Biobehavioral Reviews*, 2020.

J. P. A. Ioannidis. Why most published research findings are false. *PLoS Medicine*, 2(8):e124, 2005.

N. Japkowicz and M. Shah. Performance evaluation in machine learning. In *Machine Learning in Radiation Oncology*, pages 41–56. Springer, 2015.

L. Joskowicz, D. Cohen, N. Caplan, and J. Sosna. Inter-observer variability of manual contour delineation of structures in CT. *European Radiology*, 29(3):1391–1399, 2019.

S. Kakarmath, A. Esteva, R. Arnaout, H. Harvey, S. Kumar, E. Muse, F. Dong, L. Wedlund, and J. Kvedar. Best practices for authors of healthcare-related artificial intelligence manuscripts. *NPJ Digital Medicine*, 3:134–134, 2020.

P. Kellmeyer. Ethical and legal implications of the methodological crisis in neuroimaging. *Cambridge Quarterly of Healthcare Ethics*, 26(4):530–554, 2017.

N. L. Kerr. HARKing: hypothesizing after the results are known. *Personality and social psychology review*, 2(3):196–217, 1998.

P. Langley. The changing science of machine learning. *Machine Learning*, 82:275–279, 2011.

A. J. Larrazabal, N. Nieto, V. Peterson, D. H. Milone, and E. Ferrante. Gender imbalance in medical imaging datasets produces biased classifiers for computer-aided diagnosis. *Proceedings of the National Academy of Sciences*, 2020.

Z. C. Lipton and J. Steinhardt. Troubling trends in machine learning scholarship: some ML papers suffer from flaws that could mislead the public and stymie future research. *Queue*, 17(1):45–77, 2019.

G. Litjens, T. Kooi, B. E. Bejnordi, A. A. A. Setio, F. Ciompi, M. Ghafoorian, J. A. van der Laak, B. Van Ginneken, and C. I. Sánchez. A survey on deep learning in medical image analysis. *Medical Image Analysis*, 42:60–88, 2017.

X. Liu, L. Faes, A. U. Kale, S. K. Wagner, D. J. Fu, A. Bruynseels, T. Mahendiran, G. Moraes, M. Shamdas, C. Kern, et al. A comparison of deep learning performance against health-care professionals in detecting diseases from medical imaging: a systematic review and meta-analysis. *The Lancet Digital Health*, 2019.

L. Maier-Hein, M. Eisenmann, A. Reinke, S. Onogur, M. Stankovic, P. Scholz, T. Arbel, H. Bogunovic, A. P. Bradley, A. Carass, et al. Why rankings of biomedical image analysis competitions should be interpreted with care. *Nature Communications*, 9(1):5217, 2018a.

L. Maier-Hein, M. Eisenmann, A. Reinke, S. Onogur, M. Stankovic, P. Scholz, T. Arbel, H. Bogunovic, A. P. Bradley, A. Carass, et al. Is the winner really the best? a critical analysis of common research practice in biomedical image analysis competitions. *Nature Communications*, 2018b.

M. Mitchell, S. Wu, A. Zaldivar, P. Barnes, L. Vasserman, B. Hutchinson, E. Spitzer, I. D. Raji, and T. Gebru. Model cards for model reporting. In *Fairness, Accountability, and Transparency (FAccT)*, pages 220–229. ACM, 2019.

A. Mori and M. Taylor. Dimensions metrics API reference & getting started. *Digital Science & Research solutions*, 2018.

S. G. Mueller, M. W. Weiner, L. J. Thal, R. C. Petersen, C. R. Jack, W. Jagust, J. Q. Trojanowski, A. W. Toga, and L. Beckett. Ways toward an early diagnosis in Alzheimer's disease: the Alzheimer's Disease Neuroimaging Initiative (ADNI). *Alzheimer's & Dementia*, 1(1):55–66, 2005.

K. Musgrave, S. Belongie, and S.-N. Lim. A metric learning reality check. In *European Conference on Computer Vision*, pages 681–699. Springer, 2020.

B. Norgeot, G. Quer, B. K. Beaulieu-Jones, A. Torkamani, R. Dias, M. Gianfrancesco, R. Arnaout, I. S. Kohane, S. Saria, E. Topol, et al. Minimum information about clinical artificial intelligence modeling: the MI-CLAIM checklist. *Nature Medicine*, 26(9):1320–1324, 2020.

L. Oakden-Rayner. Exploring large-scale public medical image datasets. *Academic Radiology*, 27(1): 106–112, 2020.

L. Oakden-Rayner, J. Dunnmon, G. Carneiro, and C. Ré. Hidden stratification causes clinically meaningful failures in machine learning for medical imaging. In *ACM Conference on Health, Inference, and Learning*, pages 151–159, 2020.

A. Oliver, A. Odena, C. Raffel, E. D. Cubuk, and I. J. Goodfellow. Realistic evaluation of semi-supervised learning algorithms. In *Neural Information Processing Systems (NeurIPS)*, 2018.

S. N. Ørting, A. Doyle, A. van Hilten, M. Hirth, O. Inel, C. R. Madan, P. Mavridis, H. Spiers, and V. Cheplygina. A survey of crowdsourcing in medical image analysis. *Human Computation*, 7:1–26, 2020.

S. H. Park and K. Han. Methodologic guide for evaluating clinical performance and effect of artificial intelligence technology for medical diagnosis and prediction. *Radiology*, 286(3):800–809, 2018.

H. V. Pham, S. Qian, J. Wang, T. Lutellier, J. Rosenthal, L. Tan, Y. Yu, and N. Nagappan. Problems and opportunities in training deep learning software systems: an analysis of variance. In *IEEE/ACM International Conference on Automated Software Engineering*, pages 771–783, 2020.

R. A. Poldrack, G. Huckins, and G. Varoquaux. Establishment of best practices for evidence for prediction: a review. *JAMA Psychiatry*, 77(5):534–540, 2020.

E. H. Pooch, P. L. Ballester, and R. C. Barros. Can we trust deep learning models diagnosis? the impact of domain shift in chest radiograph classification. In *MICCAI workshop on Thoracic Image Analysis*. Springer, 2019.

A. A. Pulini, W. T. Kerr, S. K. Loo, and A. Lenartowicz. Classification accuracy of neuroimaging biomarkers in attention-deficit/hyperactivity disorder: Effects of sample size and circular analysis. *Biological Psychiatry: Cognitive Neuroscience and Neuroimaging*, 4(2):108–120, 2019.

S. Rabanser, S. Günnemann, and Z. C. Lipton. Failing loudly: an empirical study of methods for detecting dataset shift. In *Neural Information Processing Systems (NeurIPS)*. 2018.

T. Rädsch, S. Eckhardt, F. Leiser, K. D. Pandl, S. Thiebes, and A. Sunyaev. What your radiologist might be missing: using machine learning to identify mislabeled instances of X-ray images. In *Hawaii International Conference on System Sciences (HICSS)*. 2020.

A. N. Richter and T. M. Khoshgoftaar. Sample size determination for biomedical big data with limited labels. *Network Modeling Analysis in Health Informatics and Bioinformatics*, 9(1):12, 2020.

R. D. Riley, T. P. Debray, G. S. Collins, L. Archer, J. Ensor, M. van Smeden, and K. I. Snell. Minimum sample size for external validation of a clinical prediction model with a binary outcome. *Statistics in Medicine*, 2021.

M. Roberts, D. Driggs, M. Thorpe, J. Gilbey, M. Yeung, S. Ursprung, A. I. Aviles-Rivero, C. Etmann, C. McCague, L. Beer, et al. Common pitfalls and recommendations for using machine learning to detect and prognosticate for COVID-19 using chest radiographs and CT scans. *Nature Machine Intelligence*, 3(3):199–217, 2021.

R. Roelofs, V. Shankar, B. Recht, S. Fridovich-Keil, M. Hardt, J. Miller, and L. Schmidt. A meta-analysis of overfitting in machine learning. In *Neural Information Processing Systems (NeurIPS)*, pages 9179–9189, 2019.

T. Rohlfing. Image similarity and tissue overlaps as surrogates for image registration accuracy: widely used but unreliable. *IEEE Transactions on Medical Imaging*, 31(2):153–163, 2011.

R. Rosenthal. The file drawer problem and tolerance for null results. *Psychological Bulletin*, 86(3):638, 1979.

S. Saeb, L. Lonini, A. Jayaraman, D. C. Mohr, and K. P. Kording. The need to approximate the use-case in clinical machine learning. *Gigascience*, 6(5):gix019, 2017.

K. Sakai and K. Yamada. Machine learning studies on major brain diseases: 5-year trends of 2014–2018. *Japanese Journal of Radiology*, 37(1):34–72, 2019.

G. Santafe, I. Inza, and J. A. Lozano. Dealing with the evaluation of supervised classification algorithms. *Artificial Intelligence Review*, 44(4):467–508, 2015.

W. B. Schwartz, R. S. Patil, and P. Szolovits. Artificial intelligence in medicine, 1987.

D. Sculley, G. Holt, D. Golovin, E. Davydov, T. Phillips, D. Ebner, V. Chaudhary, M. Young, J.-F. Crespo, and D. Dennison. Hidden technical debt in machine learning systems. In *Neural Information Processing Systems (NeurIPS)*, pages 2503–2511, 2015.

M. P. Sendak, J. D'Arcy, S. Kashyap, M. Gao, M. Nichols, K. Corey, W. Ratliff, and S. Balu. A path for translation of machine learning products into healthcare delivery. *European Medical Journal Innovations*, 10:19–00172, 2020.

V. Shankar, R. Roelofs, H. Mania, A. Fang, B. Recht, and L. Schmidt. Evaluating machine accuracy on imagenet. In *International Conference on Machine Learning (ICML)*, 2020.

A. L. Simpson, M. Antonelli, S. Bakas, M. Bilello, K. Farahani, B. Van Ginneken, A. Kopp-Schneider, B. A. Landman, G. Litjens, B. Menze, et al. A large annotated medical image dataset for the development and evaluation of segmentation algorithms. *arXiv preprint arXiv:1902.09063*, 2019.

E. W. Steyerberg and F. E. Harrell. Prediction models need appropriate internal, internal–external, and external validation. *Journal of Clinical Epidemiology*, 69:245–247, 2016.

H. Suresh and J. V. Guttag. A framework for understanding unintended consequences of machine learning. *arXiv preprint arXiv:1901.10002*, 2019.

D. Szucs and J. P. Ioannidis. Sample size evolution in neuroimaging research: an evaluation of highly-cited studies (1990-2012) and of latest practices (2017-2018) in high-impact journals. *NeuroImage*, page 117164, 2020.

T. Tasdizen, M. Sajjadi, M. Javanmardi, and N. Ramesh. Improving the robustness of convolutional networks to appearance variability in biomedical images. In *International Symposium on Biomedical Imaging (ISBI)*, pages 549–553. IEEE, 2018.

R. Tatman, J. VanderPlas, and S. Dane. A practical taxonomy of reproducibility for machine learning research. In *ICML workshop on Reproducibility in Machine Learning*, 2018.

D. Teney, K. Kafle, R. Shrestha, E. Abbasnejad, C. Kanan, and A. v. d. Hengel. On the value of out-of-distribution testing: an example of Goodhart's Law. In *Neural Information Processing Systems (NeurIPS)*, 2020.

R. Thomas and D. Uminsky. The problem with metrics is a fundamental problem for AI. *arXiv preprint arXiv:2002.08512*, 2020.

W. H. Thompson, J. Wright, P. G. Bissett, and R. A. Poldrack. Meta-research: Dataset decay and the problem of sequential analyses on open datasets. *eLife*, 9:e53498, 2020.

E. J. Topol. High-performance medicine: the convergence of human and artificial intelligence. *Nature Medicine*, 25(1):44–56, 2019.

A. Torralba and A. A. Efros. Unbiased look at dataset bias. In *Computer Vision and Pattern Recognition (CVPR)*, pages 1521–1528, 2011.

B. Van Calster, L. Wynants, J. F. Verbeek, J. Y. Verbakel, E. Christodoulou, A. J. Vickers, M. J. Roobol, and E. W. Steyerberg. Reporting and interpreting decision curve analysis: a guide for investigators. *European urology*, 74:796, 2018.

B. Van Calster, D. J. McLernon, M. Van Smeden, L. Wynants, and E. W. Steyerberg. Calibration: the Achilles heel of predictive analytics. *BMC Medicine*, 17(1):1–7, 2019.

G. Varoquaux. Cross-validation failure: small sample sizes lead to large error bars. *NeuroImage*, 180: 68–77, 2018.

M. Voets, K. Møllersen, and L. A. Bongo. Replication study: Development and validation of deep learning algorithm for detection of diabetic retinopathy in retinal fundus photographs. *arXiv preprint arXiv:1803.04337*, 2018.

C. Wachinger, A. Rieckmann, S. Pölsterl, A. D. N. Initiative, et al. Detect and correct bias in multi-site neuroimaging datasets. *Medical Image Analysis*, 67:101879, 2021.

K. L. Wagstaff. Machine learning that matters. In *International Conference on Machine Learning (ICML)*, pages 529–536, 2012.

J. Wen, E. Thibeau-Sutre, M. Diaz-Melo, J. Samper-González, A. Routier, S. Bottani, D. Dormont, S. Durrleman, N. Burgos, O. Colliot, et al. Convolutional neural networks for classification of Alzheimer's disease: overview and reproducible evaluation. *Medical Image Analysis*, page 101694, 2020.

K. Whitaker and O. Guest. #bropenscience is broken science. *The Psychologist*, 33:34–37, 2020.

J. Wilkinson, K. F. Arnold, E. J. Murray, M. van Smeden, K. Carr, R. Sippy, M. de Kamps, A. Beam, S. Konigorski, C. Lippert, et al. Time to reality check the promises of machine learning-powered precision medicine. *The Lancet Digital Health*, 2020.

M. J. Willemink, W. A. Koszek, C. Hardell, J. Wu, D. Fleischmann, H. Harvey, L. R. Folio, R. M. Summers, D. L. Rubin, and M. P. Lungren. Preparing medical imaging data for machine learning. *Radiology*, page 192224, 2020.

J. K. Winkler, C. Fink, F. Toberer, A. Enk, T. Deinlein, R. Hofmann-Wellenhof, L. Thomas, A. Lallas, A. Blum, W. Stolz, et al. Association between surgical skin markings in dermoscopic images and diagnostic performance of a deep learning convolutional neural network for melanoma recognition. *JAMA Dermatology*, 155(10):1135–1141, 2019.

R. F. Wolff, K. G. Moons, R. D. Riley, P. F. Whiting, M. Westwood, G. S. Collins, J. B. Reitsma, J. Kleijnen, and S. Mallett. Probast: a tool to assess the risk of bias and applicability of prediction model studies. *Annals of internal medicine*, 170(1):51–58, 2019.

C.-W. Woo, L. J. Chang, M. A. Lindquist, and T. D. Wager. Building better biomarkers: brain models in translational neuroimaging. *Nature Neuroscience*, 20(3):365, 2017.

X. Yu, H. Zheng, C. Liu, Y. Huang, and X. Ding. Classify epithelium-stroma in histopathological images based on deep transferable network. *Journal of Microscopy*, 271(2):164–173, 2018.

J. R. Zech, M. A. Badgeley, M. Liu, A. B. Costa, J. J. Titano, and E. K. Oermann. Variable generalization performance of a deep learning model to detect pneumonia in chest radiographs: a cross-sectional study. *PLoS Medicine*, 15(11):e1002683, 2018.

O. Zendel, M. Murschitz, M. Humenberger, and W. Herzner. How good is my test data? introducing safety analysis for computer vision. *International Journal of Computer Vision*, 125(1-3):95–109, 2017.

S. K. Zhou, H. Greenspan, C. Davatzikos, J. S. Duncan, B. van Ginneken, A. Madabhushi, J. L. Prince, D. Rueckert, and R. M. Summers. A review of deep learning in medical imaging: Image traits, technology trends, case studies with progress highlights, and future promises. *Proceedings of the IEEE*, pages 1–19, 2020.

Appendix

A. *Testing procedures for predictive models*

There is no one-size-fit-all testing procedures for machine learning classifiers. However, in this section we provide some recommendations on what to (not) do via an illustrative example.

Suppose we are interested in detecting cancer from lung images. Given a dataset of healthy and cancerous images, and a performance metric of interest –such as accuracy– how can we design statistically-sound evaluation of a classifier? There are several different underlying questions that call for different methods.

1) Evaluating a prediction rule: The first question that we might be interested in is: given a prediction rule how well does it perform? The prediction rule can be independent of the images, or it can come from the output of a classifier trained on the data. In both settings, evidence for clinical application of the prediction rule, for instance as required by regulatory agencies, calls for statistical evaluation.

For evaluating the prediction rule we can use confidence intervals or null-hypothesis testing. For this we need test data, which (in machine learning) is often a held-out part of the existing dataset, or new data –external validation. The size of the test set then determines the statistical power: the confidence errors on the measure of the prediction performance and the effect size that can be detected. The test set should be large enough, for example too small test sets lead to large error bars of the estimated prediction performance [Varoquaux, 2018]. Riley et al. [2021] give recommendations on minimum sample sizes for various performance metrics.

2) Evaluation of a machine-learning procedure: Another question we might be interested in is to evaluate a machine-learning procedure. Unlike a prediction rule, by *machine-learning procedure* we refer the full process of starting from training data, extracting a prediction rule, and using it to classify test images as healthy or cancerous. This question is often of interest in machine-learning research, or if we want to retrain an existing prediction rule on new data. Here we need different evaluation techniques because the machine-learning procedure has several uncontrolled sources of variance, such as the training set or random initialization [Bouthillier et al., 2021]. For machine-learning research, conclusions on a given procedure should not be driven by the choice of particularly favorable training set if we cannot expect similar performance when using new training data. On the contrary, for clinical applications, it is safer to evaluate an already trained algorithm which will be used as the prediction rule in practice, to rule out the possibility of a poor performance if training the algorithm on new training data.

Given our dataset of lung images, a good evaluation of a learning procedure requires repeatedly sampling different training and testing data –as in a cross-validation loop–, as well as other sources of variance. Due to the flexibility of machine-learning classifiers, it is hard derive closed-form expressions of confidence intervals or p-values to account for all the sources of variability. Instead, we can estimate the distribution of performance scores by repeating the experiments with such variations and deduce confidence intervals [see eg procedures in Bouthillier et al., 2021]. Note that standard statistical tests (such as the t-test) cannot be used across cross-validation folds, as these are not independent samples [Varoquaux, 2018].

Sample size is an important factor to the success of prediction studies, both for the training data and the testing data. To evaluate of much the amount of data impacts the prediction performance, we can use

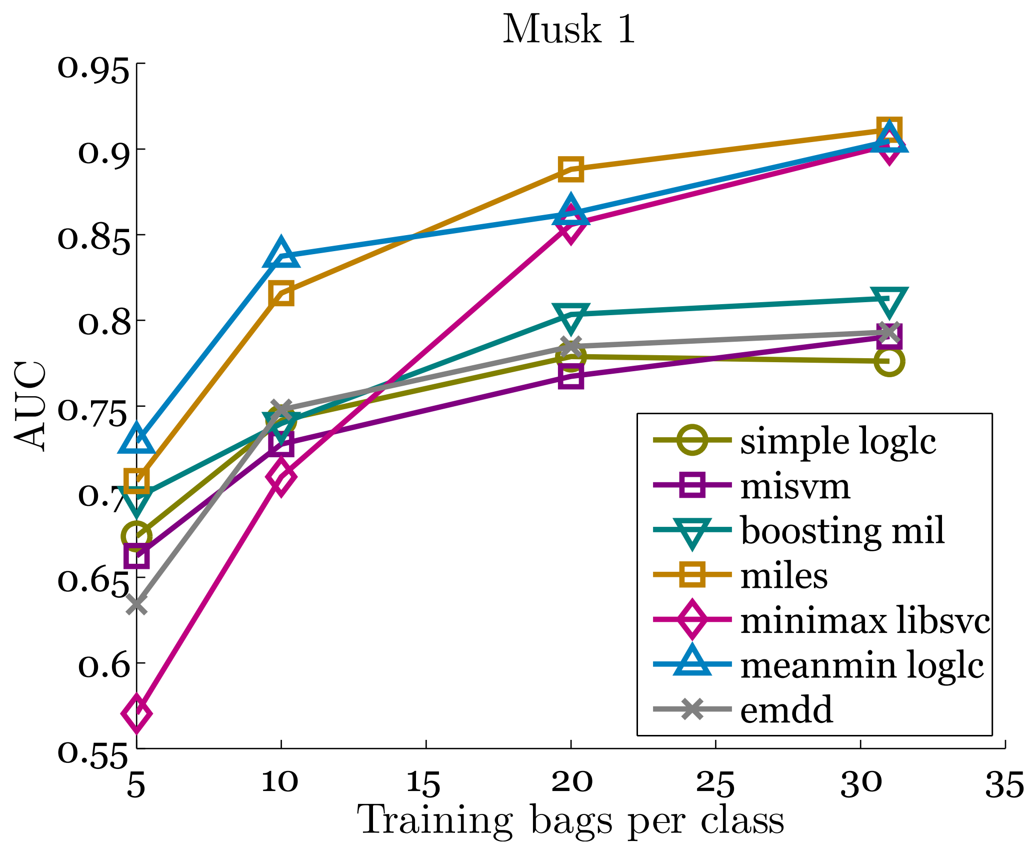

Fig. 4. Learning curves (AUC performance) for Musk1 dataset for seven classifiers as a function of the training set size. Reproduced from [Cheplygina et al., 2015].

learning curves [1] where we vary the training set size, evaluate the trained classifier on the test set, and plot the performance metric as a function of the training set size. If the curve is flattening, we might conclude that adding more training data will not improve performance. We might also be able to observe that less flexible classifiers (such as linear models) might outperform more flexible classifiers (such as neural networks) when the training set is small, but the situation to reverse when more training data is added. To give a concrete example, we use results from a machine-learning paper by one of the authors [Cheplygina et al., 2015] where classifiers are evaluated on non-medical datasets, but which with similar dataset sizes and evaluation metrics as often used in medical imaging. Fig. 4 shows an example for the dataset called for Musk1, and for seven classifiers, represented by different lines in the plot. We see that the area under the curve (AUC, higher is better) increases with the training set size, but the slopes of the classifiers are different. For example, "minimax libsvc" starts out being the worst classifier, but is among the best at larger training sizes. Ideally, this plot should have also included error bars on the performances.

3) Comparing machine-learning procedures: In machine-learning research we might want to evaluate that a classifier is better than one or more competing classifiers. The question is then whether the difference in the observed performance metrics is due to chance.

Given a particular dataset and a classifier –a learning procedure–, cross-validation can give an estimate

[1]Note that we use the original definition of learning curves where a classifier is trained and evaluated multiple times, rather than the recent trend of referring to the loss of a single training run

of the expected performance and its distribution. But we cannot yet conclude our classifier is better than another classifier for detecting lung cancer in images in general: in particular, we would need to evaluate the classifier on other, independent, datasets.

In this scenario, we can compare *ranks* of classifiers on multiple independent datasets to conclude that a classifier is generally better than another, as recommended by Demšar [2006] (though with caveats pointed out by the same author [Demšar, 2008]). Based on the number of datasets (samples) and the number of classifiers, we can test whether the average classifier ranks are due to chance. If not, we can use a post-hoc test to find the critical difference: the minimum difference in ranks that classifiers need have, to be considered significantly-different. The critical difference decreases with the number of datasets, but increases with the number of classifiers.

We show an illustration of the evaluation procedure in Table I, also based on data from results from [Cheplygina et al., 2015]. The table shows results for 14 different datasets (rows) and six classifiers (columns). For each dataset/classifier combination, the mean and standard error of the performance metric, which is the area under the curve (AUC), is reported (missing results are due classifiers failing to converge, and are ranked as last). The last row the table shows the average ranks of the classifiers, based on the Friedman test recommended by [Demšar, 2006]. Since the null hypothesis (that the differences in these ranks overall are due to chance) is rejected, the critical difference is calculated, which for 14 datasets and six classifiers is equal to 2.0153. From these results we could conclude that although MInD is the classifier with the lowest rank, MILES and Minimax are not significantly different, because their ranks are within the critical difference from 1.7857.

TABLE I

AREA UNDER THE CURVE (AUC) AND STANDARD ERROR ($\times 100$), 5×10-FOLD CROSS-VALIDATION FOR 14 DATASETS AND 6 CLASSIFIERS. THE LAST ROW SHOWS THE CLASSIFIER RANKS FROM THE FRIEDMAN TEST, FOR WHICH THE CRITICAL DIFFERENCE IS 2.0153. CLASSIFIERS IN BOLD ARE BEST, OR NOT SIGNIFICANTLY WORSE THAN BEST. REPRODUCED FROM [CHEPLYGINA ET AL., 2015].

	Classifier					
Data	emdd	misvm	boosting	miles	minimax	meanmin
Musk1	87.4 (2.1)	81.3 (2.5)	74.3 (2.6)	92.8 (1.2)	89.1 (1.9)	93.4 (1.2)
Musk2	86.9 (2.1)	81.5 (2.1)	73.6 (2.3)	95.3 (0.8)	89.0 (1.5)	95.4 (1.4)
Fox	67.6 (3.2)	53.9 (1.6)	61.1 (1.9)	69.8 (1.7)	58.1 (1.3)	60.5 (1.9)
Tiger	75.4 (2.9)	83.3 (1.3)	84.1 (1.6)	87.2 (1.6)	81.4 (1.3)	85.1 (1.7)
Elephant	88.5 (2.1)	84.1 (1.4)	89.0 (1.4)	88.3 (1.3)	88.2 (1.0)	93.1 (0.8)
African	91.5 (1.0)	63.4 (1.2)	88.9 (0.9)	58.9 (1.7)	84.5 (1.5)	96.7 (0.4)
Beach	84.7 (1.3)	49.6 (1.6)	85.0 (1.1)	60.0 (1.9)	82.4 (0.9)	92.3 (0.6)
AjaxOrange	-	93.6 (1.1)	97.9 (0.5)	-	91.1 (0.9)	98.6 (0.4)
alt.atheism	51.0 (5.2)	70.9 (2.6)	-	47.1 (2.4)	80.6 (1.8)	94.9 (1.0)
comp.graphics	48.2 (3.2)	59.3 (2.8)	56.3 (2.6)	57.2 (2.6)	57.1 (2.7)	92.2 (1.4)
BrownCreeper	94.5 (0.9)	85.8 (0.7)	95.4 (0.4)	95.8 (0.3)	94.1 (0.4)	95.5 (0.3)
WinterWren	98.5 (0.3)	95.3 (0.4)	97.0 (1.5)	99.2 (0.2)	98.1 (0.2)	99.5 (0.1)
Web1	-	89.7	77.8 (5.7)	88.2 (4.7)	90.4	76.0 (2.7)
Web4	60.6 (1.1)	81.2	61.8 (4.9)	70.8 (1.6)	86.7	73.7 (3.2)
Rank	4.1786	4.3571	3.9286	**3.1786**	**3.5714**	**1.7857**

TABLE II
REGRESSION ANALYSIS OF PUBLISHED ACCURACY AS A FUNCTION OF SAMPLE SIZE AND PUBLICATION YEAR

	coef	Confidence interval			p value
pMCI vs sMCI ($n = 166$)					
log(subjects)	-0.0352	-0.059	—	-0.012	0.004
Year	0.0074	0.002	—	0.012	0.004
AD vs HC ($n = 86$)					
log(subjects)	-0.0188	-0.037	—	-0.000	0.047
Year	0.0024	-0.002	—	0.006	0.224

B. *Brain imaging biomarkers meta-analysis details*

While the sample size of studies increases with time, there is a wide variability. We run a ~~multivariate~~ multivariable regression analysis, to separate out the effect of sample size of the study and publication date on reported prediction accuracy. Table II gives the corresponding estimated normalized coefficients, confidence intervals, and p-values. It confirms what is visible on fig.1c: for a given publication date, studies with larger sample sizes report lower prediction accuracy. Publication time, on the other hand, is associated with an improvement in prediction accuracy.

That reported prediction accuracy decreased with study sample size has already been reported [Varoquaux, 2018]. There are multiple reasons that can explain such a finding. First, larger cohorts tend to be more heterogeneous, and thus lead to harder prediction tasks. Second, the smaller the cohort, the smaller the test set; as a result, it is more likely that a good prediction accuracy is observed by chance, due to sampling error on the test set. This good prediction accuracy would however be misleading, as it would not reflect an actual generalization capacity to new data.

C. *Literature popularity review methods*

We give here the methodological details behind figure 2. To assess relative popularity of studies on breast versus lung cancer in medical and AI research, we quantify the prevalence of these topics in the corresponding literatures. For this, we use the Dimensions.AI app [Mori and Taylor, 2018], querying the titles and abstracts of papers, with the following two queries:

- lung AND (tumor OR nodule) AND (scan OR image)
- breast AND (tumor OR nodule) AND (scan OR image)

We do this for two categories, which are the largest subcategories within top-level categories “medical sciences” and “information computing”:

- 1112 Oncology and Carcinogenesis
- 0801 Artificial Intelligence and Image Processing

We then normalize the number of papers per year, by the total number of papers for the “cancer AND (scan OR image)” query in the respective categories (1112 Oncology or 0801 AI).

D. *Details on Kaggle challenges studied*

We selected ~~four~~ 8 medical-imaging challenges from Kaggle, which allows efficient retrieval of public and private leaderboard scores. In July ~~2020~~2021, there were around ~~10~~ 15 medical-imaging challenges available, of which we selected four based on their varying focus (classification or segmentation) and

Description	URL	Incentive	Test size	Entries
Lung cancer detection in CT scans	https://www.kaggle.com/c/data-science-bowl-2017	1M USD	max 1K	394
Schizophrenia classification in MR scans	https://www.kaggle.com/c/mlsp-2014-mri/overview	Publications	120	313
Lung pneumothorax segmentation in X-rays	https://www.kaggle.com/c/siim-acr-pneumothorax-segmentation	30K USD	max 6K	350
Nerve segmentation in ultrasound images	https://www.kaggle.com/c/ultrasound-nerve-segmentation	100K USD	5.5K	922
Intracranial hemorrhage detection in CT images	https://www.kaggle.com/c/rsna-intracranial-hemorrhage-detection	15K USD	120K	2 553
Prostate cancer grade assessment	https://www.kaggle.com/c/prostate-cancer-grade-assessment	25K USD	1K	19 723
COVID-19 abnormalities location on chest radiographs	https://www.kaggle.com/c/siim-covid19-detection	100K USD	1 200	32 307
Pneumonia detection from chest radiographs	https://www.kaggle.com/c/rsna-pneumonia-detection-challenge	30K USD	3 00	2 001

TABLE III
DETAILS OF KAGGLE CHALLENGES USED FOR OUR ANALYSIS. THE TEST SIZE SHOWS THE NUMBER OF TEST IMAGES PROVIDED, AND THE NUMBER OF ENTRIES CORRESPONDS TO THE NUMBER OF RESULTS ON THE PRIVATE LEADERBOARD.

incentives. Table III gives details on the challenges we use to compare performance gains to evaluation noise (subsection IV-A).

For each competition, we looked at the public and private leaderboards, extracting the following information:

- Differences d_i, defined by the difference of the i-th algorithm between the public and private leaderboard
- Distribution of d_i's per competition, its mean and standard deviation
- The interval t_{10}, defined by the difference between the best algorithm, and the "top 10%" algorithm
