# Supplementary material for: Machine learning for medical imaging: methodological failures and recommendations for the future
Source: NPJ Digit Med. 2022 Apr 12;5:48. doi: 10.1038/s41746-022-00592-y (PMC9005663; doi:10.1038/s41746-022-00592-y)
Supplement: Supplementary file 2 — LaTeX source files [file 41746_2022_592_MOESM2_ESM.zip › figures/kaggle_fig.pdf]

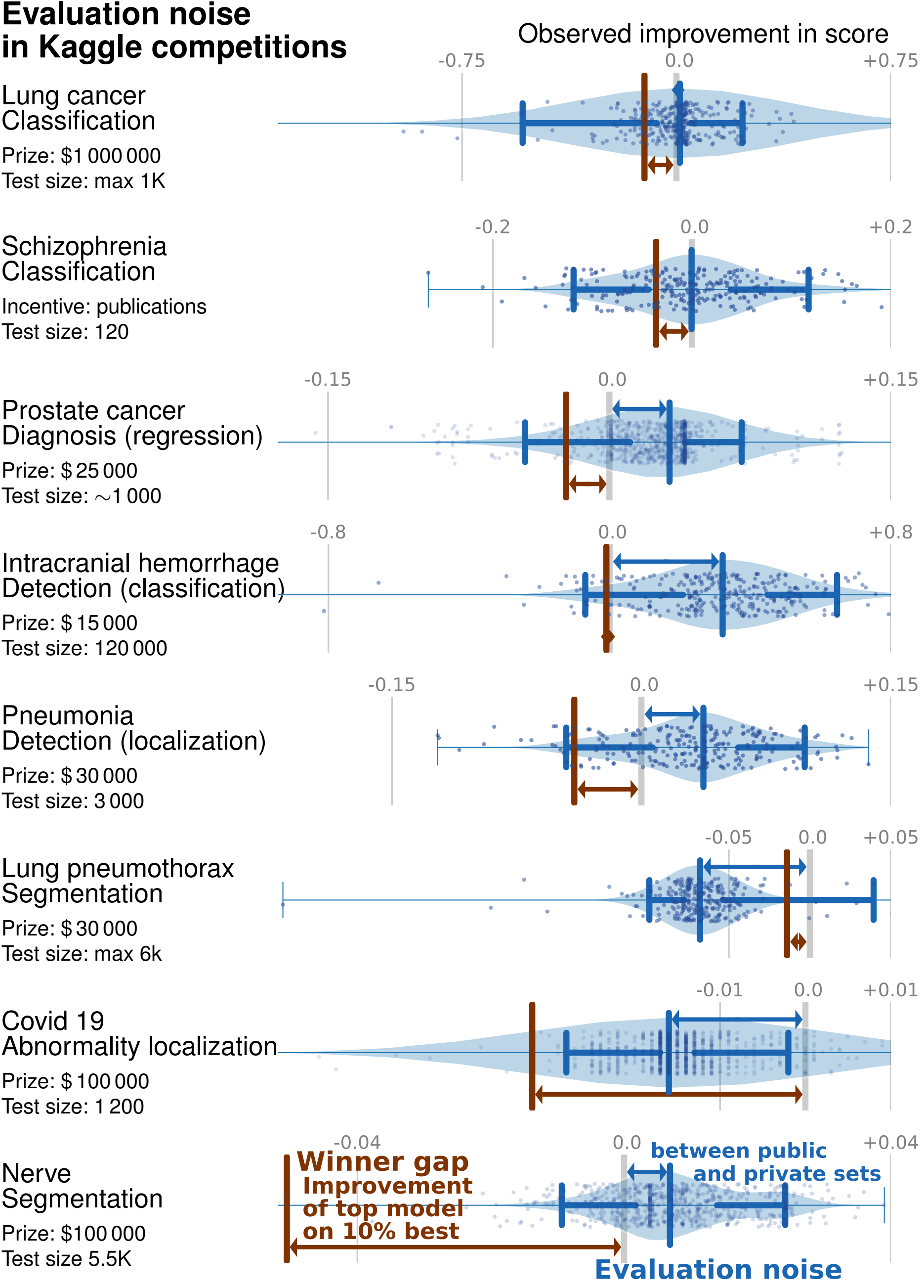

Evaluation noise
in Kaggle competitions
Observed improvement in score
Lung cancer
Classification
Prize: $1 000 000
Test size: max 1K
-0.75
0.0
+0.75
Schizophrenia
Classification
Incentive: publications
Test size: 120
-0.2
0.0
+0.2
Prostate cancer
Diagnosis (regression)
Prize: $ 25 000
Test size: ∼1 000
-0.15
0.0
+0.15
Intracranial hemorrhage
Detection (classification)
Prize: $ 15 000
Test size: 120 000
-0.8
0.0
+0.8
Pneumonia
Detection (localization)
Prize: $ 30 000
Test size: 3 000
-0.15
0.0
+0.15
Lung pneumothorax
Segmentation
Prize: $ 30 000
Test size: max 6k
-0.05
0.0
+0.05
Covid 19
Abnormality localization
Prize: $ 100 000
Test size: 1 200
-0.01
0.0
+0.01
Nerve
Segmentation
Prize: $100 000
Test size 5.5K
-0.04
0.0
+0.04
Winner gap
Improvement
of top model
on 10% best
between public
and private sets
Evaluation noise
