# Supplementary material for: Machine learning for medical imaging: methodological failures and recommendations for the future
Source: NPJ Digit Med. 2022 Apr 12;5:48. doi: 10.1038/s41746-022-00592-y (PMC9005663; doi:10.1038/s41746-022-00592-y)
Supplement: Supplementary file 2 — LaTeX source files [file 41746_2022_592_MOESM2_ESM.zip › figures/subjects_vs_year.pdf]

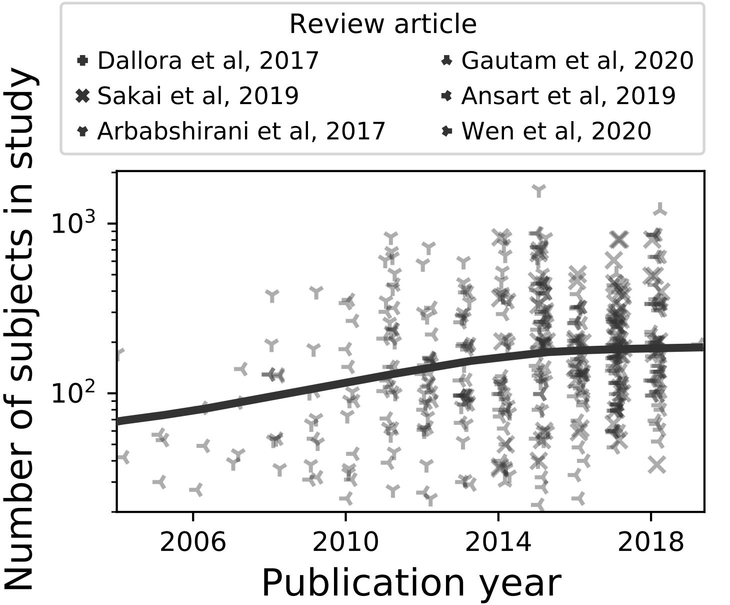

Review article
Dallora et al, 2017
Gautam et al, 2020
Sakai et al, 2019
Ansart et al, 2019
Arbabshirani et al, 2017
Wen et al, 2020
Number of subjects in study
10^3
10^2
2006
2010
2014
2018
Publication year
