# Supplementary figures and images for: Machine learning for medical imaging: methodological failures and recommendations for the future
Source: NPJ Digit Med. 2022 Apr 12;5:48. doi: 10.1038/s41746-022-00592-y (PMC9005663; doi:10.1038/s41746-022-00592-y)

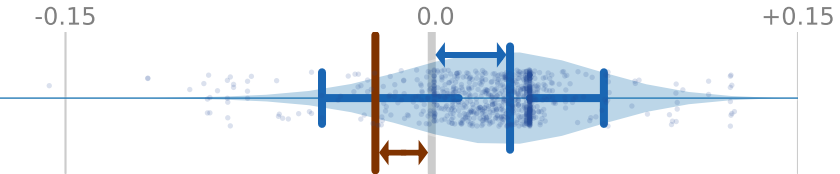

Supplement: Supplementary file 2 — LaTeX source files [file 41746_2022_592_MOESM2_ESM.zip › figures/2021-prostate_hist.pdf]

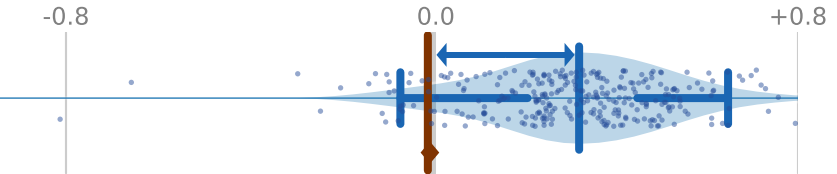

Supplement: Supplementary file 2 — LaTeX source files [file 41746_2022_592_MOESM2_ESM.zip › figures/2021-rsna-intracranial_hist.pdf]

-0.15

0.0

+0.15

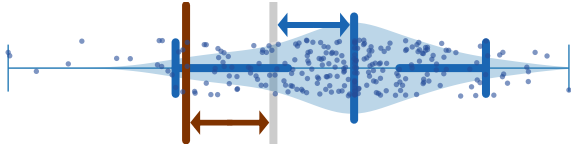

Supplement: Supplementary file 2 — LaTeX source files [file 41746_2022_592_MOESM2_ESM.zip › figures/2021-rsna-pneumonia_hist.pdf]

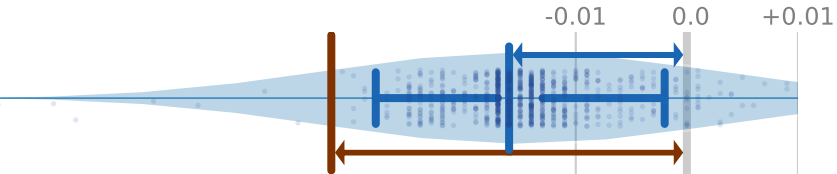

Supplement: Supplementary file 2 — LaTeX source files [file 41746_2022_592_MOESM2_ESM.zip › figures/2021-siim-covid19_hist.pdf]

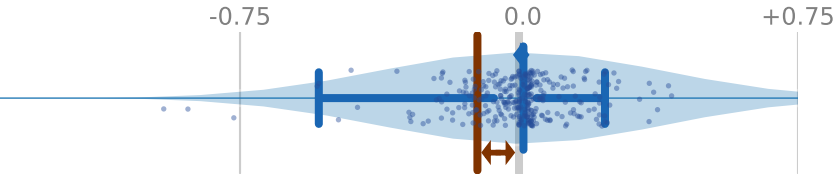

Supplement: Supplementary file 2 — LaTeX source files [file 41746_2022_592_MOESM2_ESM.zip › figures/data-science-bowl-2017_hist.pdf]

Musk 1

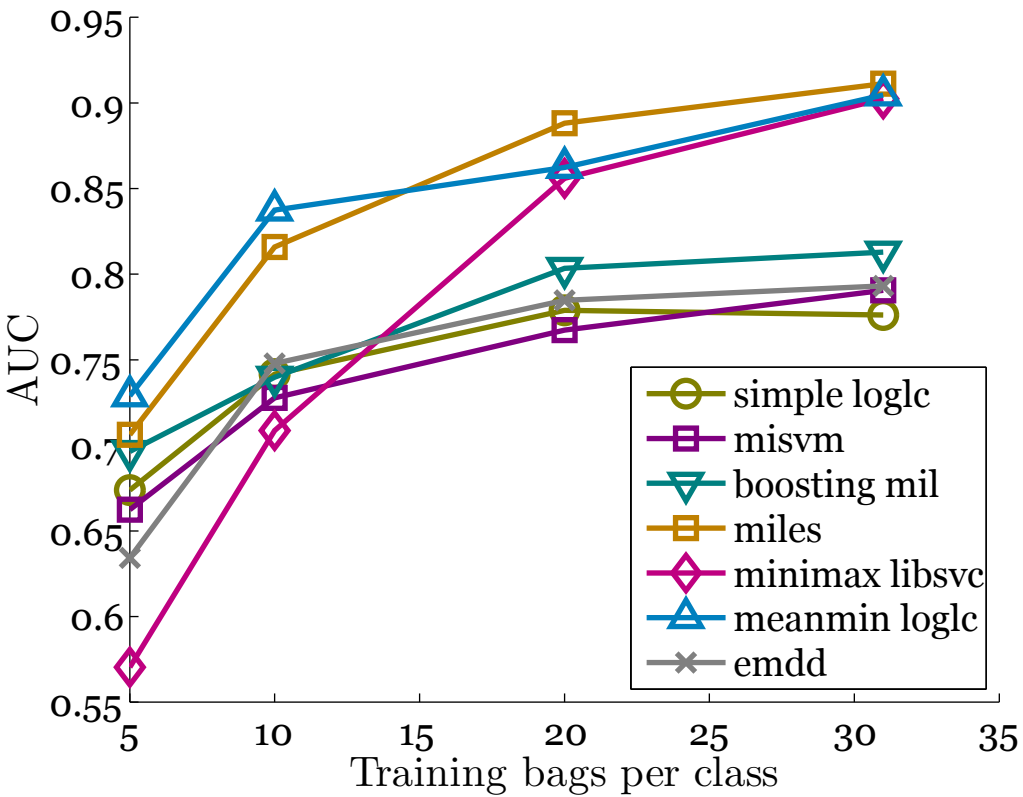

Supplement: Supplementary file 2 — LaTeX source files [file 41746_2022_592_MOESM2_ESM.zip › figures/learncurves_101.pdf]

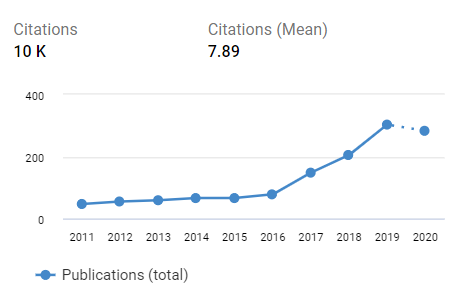

Supplement: Supplementary file 2 — LaTeX source files [file 41746_2022_592_MOESM2_ESM.zip › figures/lung_nodule_08computing.png]

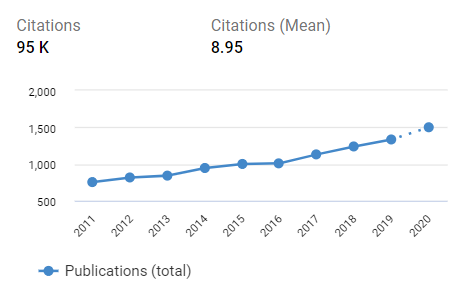

Supplement: Supplementary file 2 — LaTeX source files [file 41746_2022_592_MOESM2_ESM.zip › figures/lung_nodule_11medical.png]

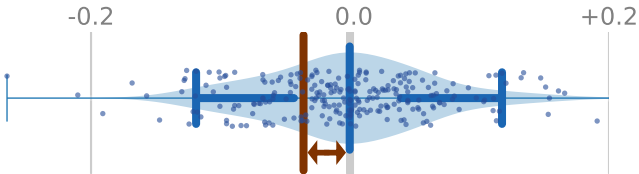

Supplement: Supplementary file 2 — LaTeX source files [file 41746_2022_592_MOESM2_ESM.zip › figures/mlsp-2014-mri_hist.pdf]

Reported prediction accuracy

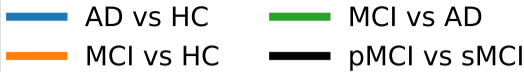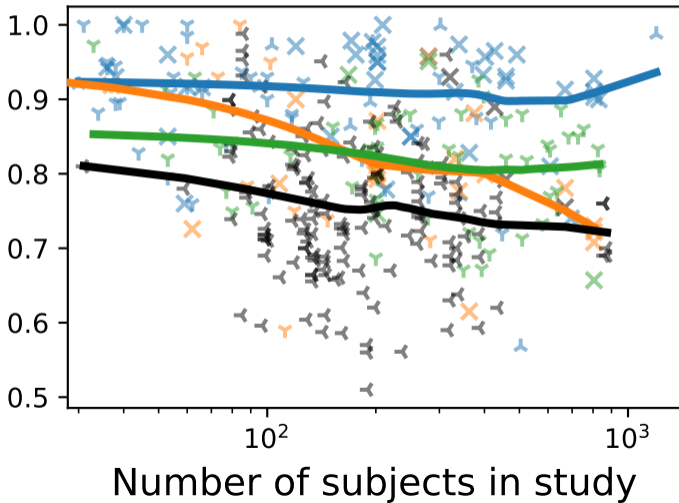

Supplement: Supplementary file 2 — LaTeX source files [file 41746_2022_592_MOESM2_ESM.zip › figures/performance_vs_subjects.pdf]

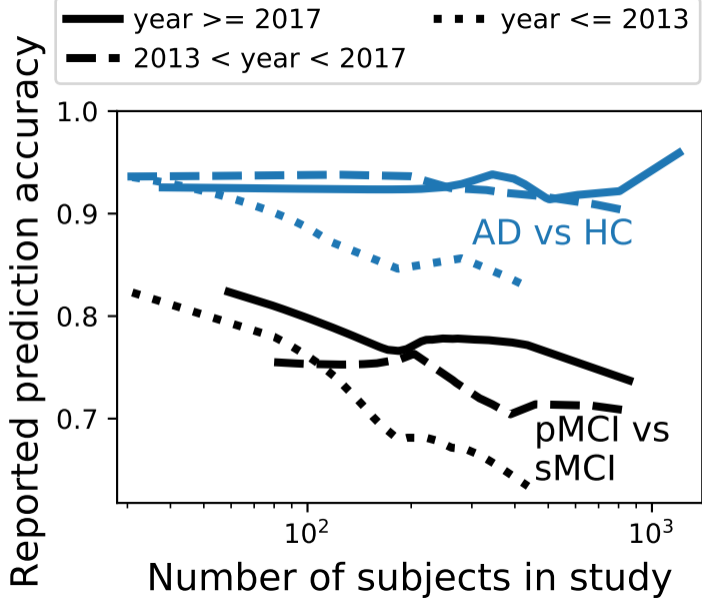

Supplement: Supplementary file 2 — LaTeX source files [file 41746_2022_592_MOESM2_ESM.zip › figures/performance_vs_subjects_time.pdf]

Reported prediction accuracy

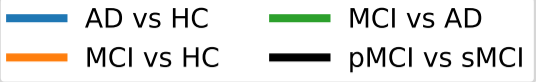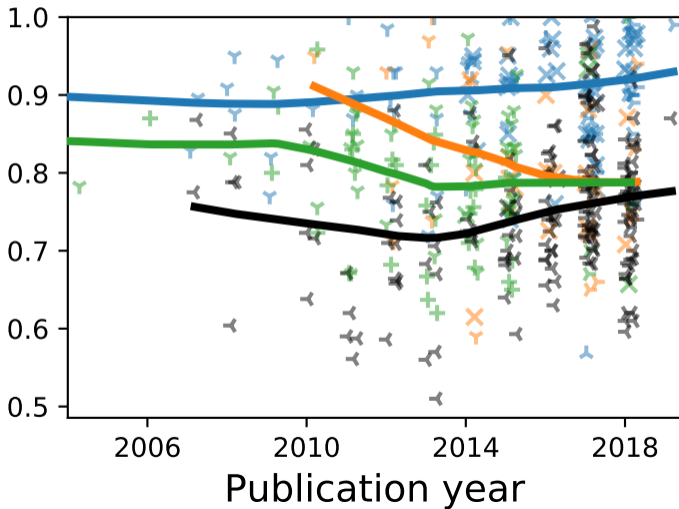

Supplement: Supplementary file 2 — LaTeX source files [file 41746_2022_592_MOESM2_ESM.zip › figures/performance_vs_year.pdf]

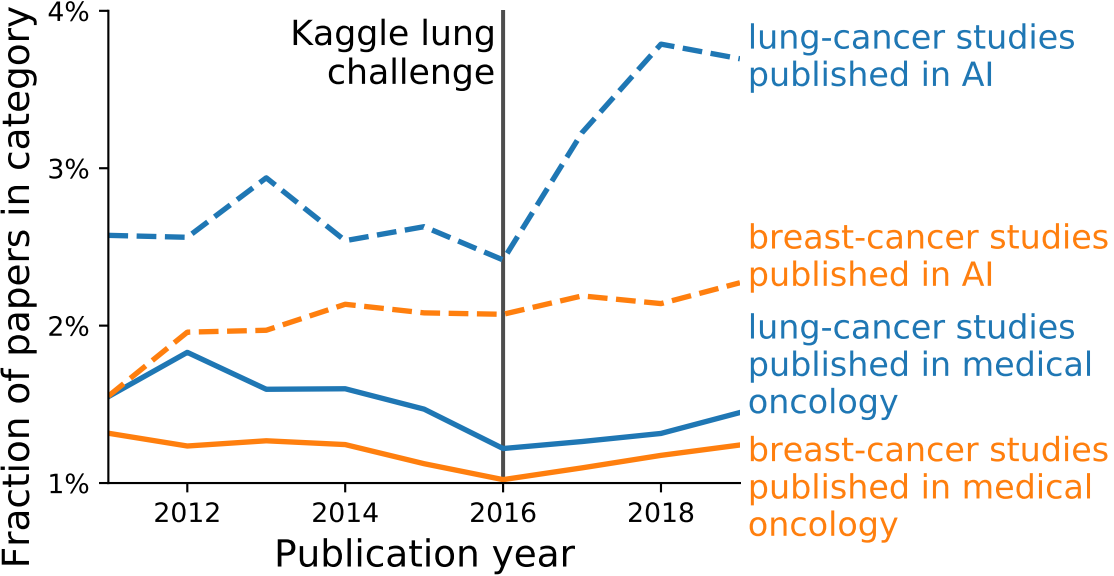

Supplement: Supplementary file 2 — LaTeX source files [file 41746_2022_592_MOESM2_ESM.zip › figures/publication_counts.pdf]

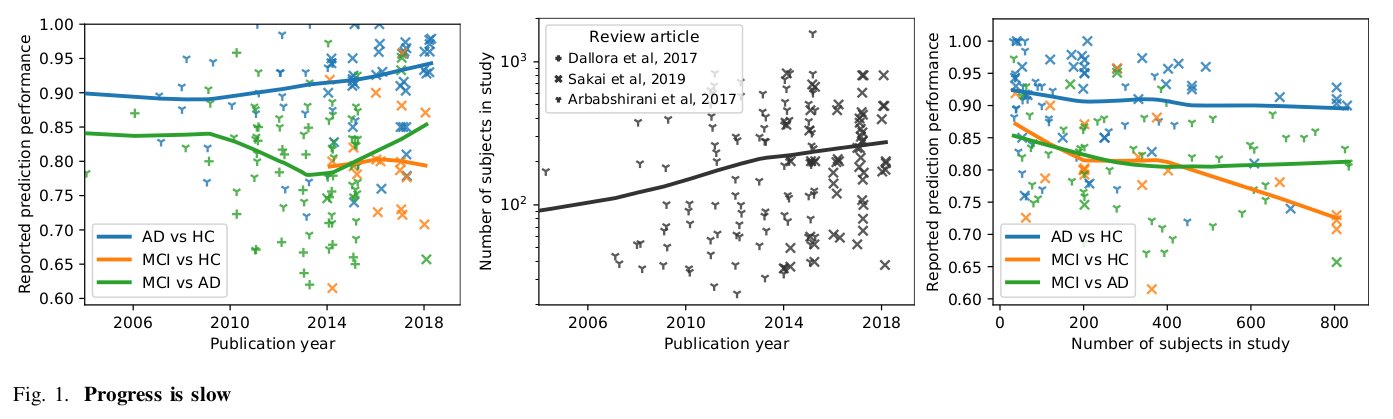

Supplement: Supplementary file 2 — LaTeX source files [file 41746_2022_592_MOESM2_ESM.zip › figures/sample_size.jpg]

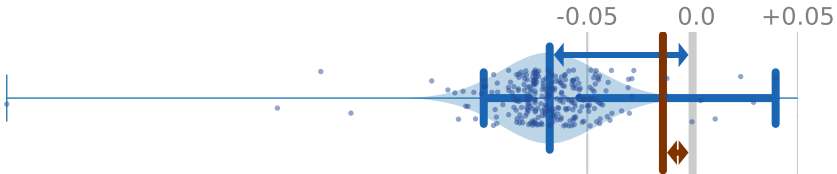

Supplement: Supplementary file 2 — LaTeX source files [file 41746_2022_592_MOESM2_ESM.zip › figures/siim-acr-pneumothorax-segmentation_hist.pdf]

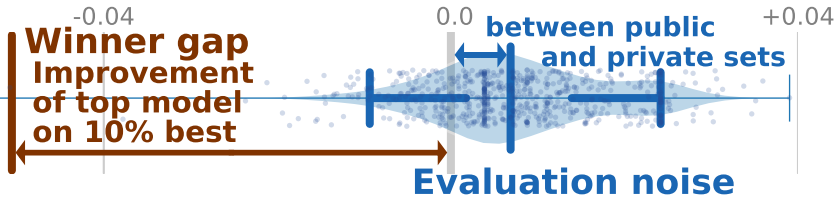

Supplement: Supplementary file 2 — LaTeX source files [file 41746_2022_592_MOESM2_ESM.zip › figures/ultrasound-nerve-segmentation_hist.pdf]
